# TGF-β1-mediated downregulation of L1CAM in pancreatic ductal adenocarcinoma drives upregulation of collagen 17A1 and MMP2, facilitating tumor invasiveness and metastasis

**DOI:** 10.1038/s41419-025-07859-8

**Published:** 2025-08-06

**Authors:** Donatella Delle Cave, Annalisa Di Domenico, Marco Fantuz, Marianna Ciotola, Maria Mangini, Silvia Buonaiuto, Brunella Corrado, Marco Corona, Federica Saracino, Gennaro Andolfi, Ilaria Di Biase, Antonio Cucciardi, Alessandro Carrer, Bruno Sainz, Teresa Pirozzi, Daniele Lo Re, Vincenza Colonna, Gabriella Minchiotti, Anna Chiara De Luca, Enza Lonardo

**Affiliations:** 1 Institute of Genetics and Biophysics ‘Adriano Buzzati-Traverso’ (IGB), CNR, Naples, Italy; 2 Department of Biology, University of Padova, Padova, Italy; 3 Veneto Institute of Molecular Medicine (VIMM), Padova, Italy; 4 Institute of Endotypes in Oncology, Metabolism and Immunology “G. Salvatore” (IEOMI), Second Unit, Naples, Italy; 5 Department of Genetics, Genomics and Informatics, University of Tennessee Health Science Center, Memphis, TN USA; 6 Interdisciplinary Research Centre on Biomaterials, University of Naples Federico II, Naples, Italy; 7 Department of Cancer, Instituto de Investigaciones Biomedicas Sols-Morreale (IIBM), CSIC-UAM, Madrid, Spain; 8 Cancer Area 3-Instituto Ramon Y Cajal de Investigacion Sanitaria (IRYCIS), Madrid, Spain; 9 Centro de Investigación Biomédica en Red, Área Cáncer, CIBERONC, ISCIII, Madrid, Spain; 10 Departamento de Química Farmacéutica y Orgánica, Facultad de Farmacia, Universidad de Granada, Granada, Spain

**Keywords:** Pancreatic cancer, Cancer microenvironment

## Abstract

The highly fibrotic microenvironment of pancreatic ductal adenocarcinoma (PDAC) poses significant challenges for effective treatment, particularly in drug delivery and tumor progression. Our study investigates the role of collagen dynamics in PDAC, revealing that TGF-β1 negatively regulates the expression of L1 cell adhesion molecule (L1CAM), leading to a more invasive tumor phenotype. We identify a subset of PDAC cells with low L1CAM expression (L1_low_) that actively influences collagen deposition and remodeling, as evidenced by the upregulation of collagen 17A1 (COL17A1) and matrix metalloproteinase 2 (MMP2), both associated with poor prognosis. In vivo studies demonstrate that L1_low_ cells correlate with increased collagen deposition, reduced sensitivity to gemcitabine, and heightened liver metastasis. The secretion of COL17A1 and MMP2 by these cells enhances their migratory capabilities and contributes to the formation of a fibrotic stroma that facilitates tumor progression. This interaction underscores the critical role of collagen in shaping the tumor microenvironment and promoting aggressive tumor behavior. Notably, treatment with Tranilast significantly reduced collagen deposition and MMP2 levels while promoting L1CAM expression, suggesting a therapeutic avenue for counteracting the aggressive characteristics of L1_low_ cells. By modulating collagen dynamics and enhancing drug delivery, Tranilast may improve treatment outcomes for patients with low L1CAM-expressing tumors. Understanding the mechanisms by which L1_low_ cells contribute to collagen secretion and tumor aggressiveness is essential for developing effective interventions in pancreatic cancer.

## Introduction

Pancreatic ductal adenocarcinoma (PDAC) is a devastating and essentially incurable disease, with an overall 5-year-survival rate of ~8% due to late diagnosis and high chemoresistance [1]. Incidence and mortality are increasing dramatically and only ~20% of pancreatic cancer patients are eligible for surgical resection. Hence, PDAC is predicted to become the second leading cause of cancer-related death by 2030 [2, 3]. PDAC is characterized by a pronounced resistance to radiation, cytotoxic and molecular-targeting therapies [4, 5]. This resistance is partially attributed to the prominent desmoplastic reaction, which consists of dense fibrotic stroma covering over 90% of the tumor volume [6]. Desmoplasia is the result of the activation and increased proliferation of myofibroblasts-like cells, in particular pancreatic stellate cells (PSCs) and cancer-associated fibroblast (CAFs), and is also accompanied by augmented deposition of different extracellular matrix (ECM) proteins, such as collagen, hyaluronans, laminins and fibronectin [7–9]. Fibrillar collagen, particularly collagen I and IV, are the most abundant ECM components, accounting for up to 80% of ECM mass. Fibrillar collagen has been considered pro-tumorigenic: a scaffold and reservoir for soluble growth factors that serve as a nutrient source for cancer cells and a physical barrier that increases chemoresistance [10–13]. Furthermore, fiber collagen orientation, crosslinking and remodeling have emerged as promising diagnostic markers. More than 25% of the amino acids in collagen are incorporated as proline and half of them are hydroxylated by the prolyl-4-hydroxylase (P4H) within the endoplasmic reticulum. Hydroxylation of proline residues is a critical modification required to stabilize the triple helix of collagen [14]. Despite improvements in the understanding of the molecular mechanisms underlying pancreatic cancer biology and in defining new curative strategies, the therapeutic needs for this disease have not yet been fulfilled. Collagen fibrils are mostly synthesized and secreted by stromal cells, although cancer cells also deposit a small fraction of the total tumor collagen [15, 16]. Interestingly, the significance of tumor cell-derived collagen in PDAC growth, progression and ECM stiffness has been largely overlooked. Recently, we demonstrated that PDAC tumors with a more aggressive phenotype (i.e., high stroma and high TGF-β1 content) exhibit reduced levels of L1 cell adhesion molecule (L1CAM, L1) [17]. L1 was originally discovered in the nervous system due to its important function for axon guidance and cell migration [18, 19]. In the last few years many in vitro and in vivo studies have conferred to L1CAM a tumor promoting role and its expression is generally associated with poor prognosis [20–24]. Nevertheless, the role of L1 in pancreatic cancer remains unclear. Whereas there is a positive correlation between L1 expression and poorly-differentiated neuroendocrine pancreatic cancers [25], in PDAC patients, the expression of L1 is barely detectable [26–28]. Recently, we demonstrated that patients with high levels of L1 have a better outcome; thereby, L1 acts as a tumor suppressor gene for PDAC [17]. In detail, we found that PSCs secreted high levels of TGF-β1, which promotes a more stem-like and aggressive phenotype in PDAC cells through the downregulation of L1. In this study, we provide a deeper insight into the molecular mechanism underlying the role of L1 in PDAC progression, metastasis and tumor fibrosis. Transcriptome analysis identified L1CAM-induced differentially expressed gene networks associated with matrisome secretion and collagen remodeling, with the most highly induced transcript being collagen type XVII alpha 1 chain (*COL17A1)*. L1_low_-mediated collagen secretion and aggressiveness were effectively suppressed by inhibiting TGF-β1 in vitro, indicating that the loss of L1CAM empowers PDAC cells to overcome the growth and invasion constraints imposed by the extracellular matrix. Treatment of mice orthotopically injected with L1-Knocked Down PDAC cells (L1_KD_) with the small molecule inhibitor Tranilast (TRL, Rizaben®, CAS 53902-12-8), which is an approved drug for asthma and glaucoma (EMA/COMP/234320/2010), targeting TGF-β and collagen, resulted also in a notable reduction in matrix metalloproteinase 2 (MMP2) expression- an essential factor for tumor cells invasion- and effectively eliminated liver metastasis [29–31]. Notably, TRL treatment enhanced the tumor’s response to Gemcitabine by decreasing collagen and MMP2 levels while simultaneously increasing L1CAM expression. These findings reveal an ancillary role for decreased L1CAM signaling in advanced pancreatic cancer to promote extracellular matrix remodeling that enables invasive tumor growth by overcoming microenvironment-imposed proliferation restraints. An implication of these results is that TGF-β1/collagen inhibitor administration to pancreatic cancer patients might reverse the ability of pancreatic cancer cells to bypass the growth-restraining properties of tumor-associated desmoplasia.

## Materials and methods

### Cell cultures

Human primary pancreatic stellate cells (PSC, HUM-ICELL-G003) and the patient xenograft-derived primary cell line #354 (Tissue derivation: primary pancreatic tumor; Carcinoma type: pancreatic ductal adenocarcinoma) were cultured in RPMI, 10% FBS, and 50 units/ml penicillin/streptomycin [32]. The human PDAC cancer cell line L3.6pl (Tissue derivation: metastatic lymph node; Carcinoma type: adenosquamous carcinoma) was maintained in DMEM, 10% FBS, and 50 units/ml penicillin/streptomycin [32]. Their identity (annually) and *Mycoplasma* free-state (bi-weekly) were routinely tested by DNA fingerprinting using short tandem repeat (STR) profiling, and by PCR-based, MycoAlert Mycoplasma Detection Kit (Lonza, Bioscience), respectively. Each cell line was used within passage 4/5 since their thawing from originally frozen vials.

### RNA preparation and real-time quantitative PCR

Total RNAs were extracted with Eurogold TRIFAST kit (Euroclone) according to the manufacturer’s instructions. One microgram of total RNA was used for cDNA synthesis with High-Capacity reverse transcriptase (Thermofisher). Quantitative real-time PCR was performed using SYBR Green PCR master mix (Thermofisher), according to the manufacturer’s instructions. The list of utilized primers is shown in Table 1.

**Table 1:** List of primers.

Gene symbol	Forward primer (5’- > 3’)	Reverse primer (5’- > 3’)
*L1CAM*	CACTATGGCCTTGTCTGGGA	ACATACTGTGGCGAAAGGGA
*COL1A1*	ATGACCGAGACGTGTGGAAA	CAGTGTCTCCCTTGGGTCC
*COL5A2*	CCGGGTCTAGCTGGTGAAAG	TCTCCTCTAGGTCCTAACGGG
*COL7A1*	CGCCAAGAGATGAGTCAGCAC	CTCTGCAGGTAGGGCAGGGT
*COL12A1*	CCACAGGTTCAAGAGGTCCC	TGTGTTAGCCGGAACCTGGA
*COL17A1*	CTGACTTTGCTGGAGATCTGG	TAGGCCATCCCTTGCAGTAG
*MMP2*	ATAACCTGGATGCCGTCGT	AGGCACCCTTGAAGAAGTAGC
*GAPDH*	CAGGAGCGAGATCCCT	GGTGCTAAGCAGTTGGT

### Protein isolation and western blot analysis

Cells were lysed with RIPA buffer (50 mM Tris-HCl at pH 7.6, 150 mM NaCl, 1% NP-40, 0.5% sodium deoxycholate, 0.1% SDS, 5 mM EDTA plus proteases and phosphatases inhibitors) for 1 h at 4 °C. Total protein quantification was performed with Bio-Rad Protein Assay Dye Reagent concentrate. A total of 100 μg of protein was separated on 15% SDS–PAGE gels at 100 V and transferred to PVDF membranes for 2 h at 200 mA. PVDF membranes were hybridized with antibodies against human HyP (37067, Abcam), pSMAD2 (3108; Cell Signaling), SMAD2 (#5339; Cell Signaling), GAPDH (SC-32233, Santa Cruz Biotech), treated with peroxidase-conjugated goat anti-mouse or anti-rabbit Ig secondary antibody (DPVR-HRP, Immunologic), and then visualized by enhanced chemiluminescence (ECL Nova 2.0 XLS071, 2050 Cyanagen). *n* > 6.

### Lentiviral shRNA delivery

As a lentiviral shuttle backbone, we used a pLKO shRNA plasmid (Mission SIGMA). L1CAM expressing plasmids (pcDNA3.1) were obtained from Genscript (New Jersey, USA). As control we used pLKO shRNA empty expression vectors. Cells were then transduced with lentiviral particles in the presence of polybrene (8 µg/ml, Sigma). The cells were seeded at a density of 30,000 cells per well in 24 multiwell plates and allowed to adhere overnight. The next day, the cells were infected with the lentiviral particles for 6 h. Stably transduced cells were obtained using puromycin resistance (1 µg/mL).

### Flow cytometry and cell sorting

Flow cytometry analysis or flow cytometry cell sorting were performed using anti-human L1CAM-PE (A18361, Life technologies) and anti-human COL17A1 (MA5-31984, Invitrogen) followed by incubation with secondary anti-rabbit antibody (A21206, INVITROGEN). 7AAD (BD) was used for the exclusion of dead cells. Samples were analyzed by flow cytometry using a FACS Canto II (BD) and data were analyzed with FlowJo 9.2 software (Ashland, OR). Experiments were repeated a minimum of three times independently, with triplicate samples.

### Immunofluorescence

Cells were fixed in 4% paraformaldehyde (PFA) for 20 min at room temperature. After blocking with 5% bovine serum albumin in PBS-Triton 0.1%, cells were incubated with the unconjugated primary antibody: HyP (1:400 dilution), L1CAM (HPA005830, Sigma-Aldrich, 1:100 dilution), pSMAD2 (1:100 dilution) and COL17A1 (MA5-31984, Invitrogen, 1:100 dilution), overnight at 4 °C in the dark, and the day after, counterstained with a fluorescent-conjugated secondary antibody used at 1:200 dilution. The nuclei of cells were stained by incubating with DAPI (SIGMA). Images were acquired at room temperature using the LEICA DMI6000 inverted microscope (Leica, Heidelberg, Germany) on a DC 350 FX camera (Leica).

### Migration assays

Migration assays were performed using Boyden chambers (Corning). Briefly, PDAC (L1empty, L1_KD#1_ or L1^over^) cells were treated biweekly for a week with 120 µM of TRL (HY-B0195, MedChem Express) or 25 nM of BB-94 (HY-13564, MedChem Express). Then, 2.5 × 10^4^ L3.6pl cells were added to the inserts of the chamber for 22 h at 37 °C. Migrated cells were fixed in 4% PFA and stained with DAPI. The ratio of cells in the lower chamber versus total seeded cells was calculated. The Student’s *t* test assessed statistical significance.

### Conditioned media

PDAC cells were maintained in DMEM media supplemented with 0.5% FBS and 50 units/ml penicillin/streptomycin. Conditioned media was collected three days after plating, centrifuged and filtered prior to incubation for 24 h with PSC cells.

### Gelatin degradation (invasion) assay

Fluorescent-coated coverslips were prepared as described previously [33]. PDAC cells were plated on gelatin-coated coverslips in a 24-well plate and fixed after 24 and 48 h with 4% PFA (v/v) for 15 min at room temperature. Then, filamentous actin and nuclei were stained using Alexa Fluor™ 488 Phalloidin (Invitrogen) and Hoechst 33342 (Invitrogen), respectively. Images were acquired using a confocal microscope (LSM 510; Zeiss), and degradation areas were quantified using ImageJ software.

### Cell treatment

PDAC cells were seeded in a 6-well plate and after 24 h were treated with 120 µM of TRL either alone or in the presence of 10 ng/ml of recombinant TGF-β1 (rTGF-β1, 100-21, Peprotech), biweekly for a week. Cells were harvested with EDTA-trypsin, washed twice with PBS 1X, and collected by centrifugation (13,000 rpm per 5 min) for RNA or protein extraction.

### Tumor growth

All animal experiments have been approved by the local ministry (IACUC protocol n°1010/2023-PR) and were performed in the animal facility under pathogen-free conditions. Single-cell suspensions of 2.5 × 10^5^ #354 cells were subcutaneously injected into 6-week-old nude athymic CD1 male mice (Charles River Laboratories). When tumors reached 100 mm^3^ (around 15 days post-injection), mice were randomized and treated with vehicle (PBS 1X), TRL (50 mg/kg of mice), Gemcitabine (100 mg/kg of mice, HY-B0003, MedChem Express) alone or in combination, biweekly for three weeks via intraperitoneal (i.p.) injection. Tumor growth was monitored visually and by caliper bi-weekly. Tumor diameter and volume were calculated based on caliper measurements of tumor length and height using the formula: tumor volume = (length × width^2^)/2. For ethical reasons mice were sacrificed when tumors reach a maximum volume of 1500–2000 mm^3^.

### Orthotopic assay

Single-cell suspensions of 8 × 10^5^ L3.6pl (L1empty, L1_KD#1_ or L1^over^) cells were slowly injected into the pancreas of 6-week-old nude athymic CD1 male mice (Charles River Laboratories) (*n* = 4 per group). One week after the injection of tumor cells, mice were randomized based on their body weight and treated with vehicle (PBS 1X), TRL (50 mg/kg of mice), Gem (100 mg/kg of mice) alone or in combination, biweekly for three weeks via intraperitoneal (i.p.) injection. After 2 months from the injection, mice were sacrificed and pancreas and livers were excised, photographed and fixed in 4% PFA for the histological analyses.

### Hematoxylin/Eosin and picrosirius red staining in FFPE

According to standard procedures, Hematoxylin and Eosin (H&E) staining was carried out using 4-μm FFPE tissue sections. Briefly, the sections on slides were submerged into Lillie-Mayer’s hematoxylin solution (Sigma) for 10 min, then rinsed in tap water for 2 min. Subsequently, the slides were submerged into 2% alcoholic eosin solution (Sigma) for 2 min.

For Picrosirius Red staining (24901-250, Polysciences Inc.,), tumor sections were incubated with Sirius Red according to standard procedures. Briefly, sections were incubated with 0.1% Sirius Red solution dissolved in aqueous saturated picric acid for 1 hour and washed rapidly in 5% acetic acid. Slides were drained well, and sections were dehydrated through an ascending alcohol bath series (30, 50, 70, 95, and 100%, 1 min each) and cleared in xylene (Sigma) for 5 min. Finally, the slides were air-dried and mounted in Distyrene Plasticizer Xylene (D.P.X.) for histology (Sigma). Each sample was observed using a light LEICA DM6000 inverted microscope (Leica, Heidelberg, Germany) with a DC 350 FX camera (Leica).

### IHC and IF in FFPE

Immunostainings were carried out using 4-μm tissue sections according to standard procedures. For IHC, after antigen retrieval, samples were blocked with Peroxidase-Blocking Solution (Dako, S202386) for 10 min at room temperature, and primary anti-Human Nuclei Antibody (MAB4383, Sigma-Aldrich) was then incubated with samples overnight. Slides were washed with EnVision FLEX Wash Buffer (Dako, K800721), and the corresponding secondary antibody was incubated with the sample for 60 min at room temperature. Samples were developed using 3,3′-diaminobenzidine, counterstained with hematoxylin and mounted. Images were acquired using a digital image scanning (Nanozoomer 2.0HT, Hamamatsu) and cropped using NDP.view2. For IF, antibodies against human L1CAM (HPA005830, SIGMA), human COL17A1 (MA5-31984, Invitrogen), MMP2 (4022S, Cell Signaling) and murine COL1A1 (AB765P, Millepore) were used at a 1:100 dilution overnight at 4^o^C in the dark. The nuclei of the cell were stained with DAPI. Images were acquired using a Nikon A1 microscope. The area % stain represents the ratio of the summed absolute areas of staining versus the total tissue. The area % stain was analyzed by Fiji ImageJ version v3.2.28.

### Gene expression data sets and GSEA analyses

The gene expression data sets used in this study are publicly available. The TCGA dataset was downloaded from The Cancer Genome Atlas (TCGA; http://xena.ucsc.edu).

### RNA-sequencing analysis

Total RNAs were extracted with Eurogold TRIFAST kit (Euroclone) according to the manufacturer’s instructions. TruSeq stranded mRNA libraries were run on the Illumina NextSeq 500 at Genomix4Life (https://www.genomix4life.com/en/bioinformatic_technologies.html.

We generated 40 million reads per lane and paired-end reads from sequencing were trimmed from adaptors and quality checked by the sequencing service. Reads were aligned against the human reference sequence GRCh38 (hg38) from the Genome Reference Consortium using BWA (Li 2009). Read counts per gene were determined using the feature Counts method from the Subread R package (Liao 2014) based on the gene information in the gtf available here ftp://ftp.ensembl.org/pub/release-94/gtf/homo_sapiens/Homo_sapiens.GRCh38.94.gtf.gz. Gene signatures (Hallmark genesets) were downloaded from GSEA—Molecular Signature Database for Gene set enrichment analysis. RNA-seq data for L1_low_ and L1^high^ cells is available at https://www.ncbi.nlm.nih.gov/geo/query/acc.cgi?acc=GSE268948 under accession number GSE268948.

### Single cell data analysis from publicly-available datasets

For the single-cell atlas of human PDAC, the fully integrated dataset was downloaded from the Zenodo repository (https://zenodo.org/records/6024273) and loaded in R using the Seurat package [34]. Cell type characterization and annotations were retrieved from the metadata slot, while the expression of selected genes was plotted using the FeaturePlot function of Seurat. For the GSE154778 dataset, files in the 10X format (barcodes, matrix, genes tables) were downloaded from the GEO database, and loaded in R using the Seurat package [35]. Datasets from primary tumors were integrated and analyzed as previously described [36]. After analysis and clustering, ductal-like cells were identified employing a panel of published expressed genes [37]. The expression and the violin plots for selected genes were obtained using the FeaturePlot and ViolinPlot function of Seurat, respectively. Cluster 7 cellular composition was inferred using SuperCT, a supervised-classifier approach published in literature [38], which gives an accurate prediction based on an a priori learning.

### Statistical analysis

Results for continuous variables are presented as means ± standard deviation (SD) unless stated otherwise of at least three independent experiments. Treatment groups were compared with the independent samples t test. Pair-wise multiple comparisons were performed with the one-way ANOVA (two-sided) with Bonferroni adjustment. The disease-free interval of patients was calculated using the Kaplan–Meier method, and differences among subgroups were assessed by the log-rank test. Experiments were performed a minimum of three independent times and always performed in independent triplicate samples. qPCR analyses were repeated a minimum of five independent times in triplicate. *p* < 0.05 was considered statistically significant. All analyses were performed using GraphPAD Prism7. Correlation analysis were performed applying the Pearson’s correlation coefficient. The sample size for the in vivo experiments was determined based on power calculation analysis conducted using the G*Power 3.1 program.”

## Results

### *COL17A1* expression is dysregulated in L1_low_ epithelial cancer cells

**Fig. 1:**
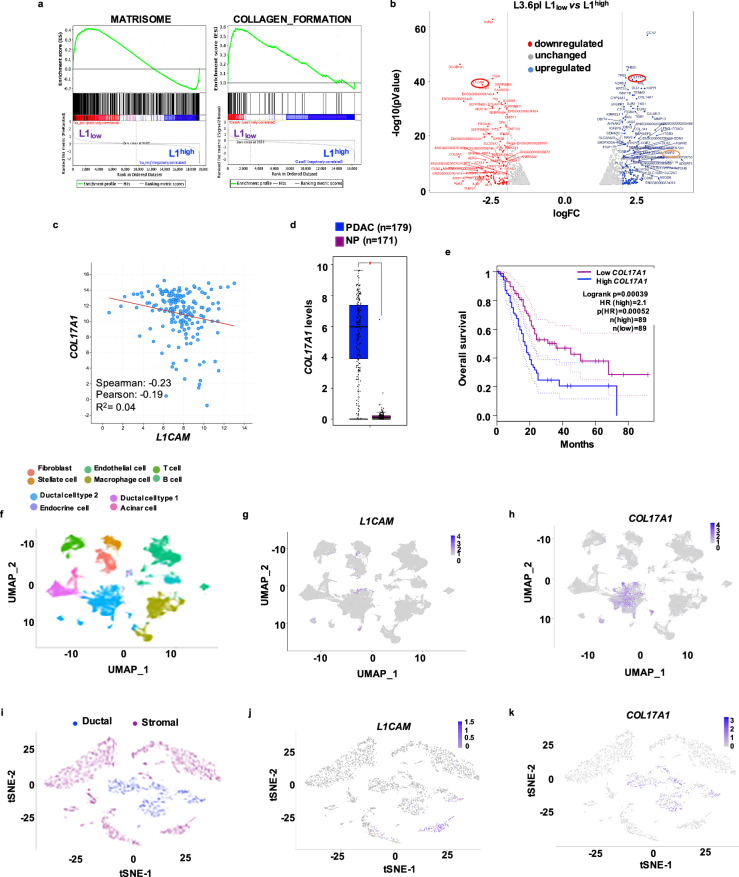
Inverse correlation between L1 and epithelial tumor cell-derived *COL17A1* associated with dismal prognosis. **a** Enrichment plot for L1_low_ *versus* L1^high^ FACS-sorted L3.6pl cells. **b** Volcano plot comparing L1_low_ *versus* L1^high^ FACS-sorted L3.6pl cells. Genes found to be differentially expressed in the RNA-seq analysis are shown in red (downregulated) and blue (upregulated). Red and orange circles denote genes of interest. **c** Inverse correlation between *COL17A1* and *L1CAM* in PDAC samples in the TCGA repository. **d** Boxplots showing the differential expression of *COL17A1* in PDAC samples *versus* normal tissue (NP) in the TCGA dataset. **e** Kaplan–Meier curves showing overall survival of PDAC patients, stratified according to the median value of *COL17A1* expression. **f** Uniform manifold approximation and projection (UMAP) of six integrated single-cell datasets derived from human pancreatic adenocarcinoma samples [27]. Clusters are color-coded according to the assigned cell type. **g** Feature plot of *L1CAM* gene expression in the PDAC atlas dataset (in **e**). Color shows average expression. **h** Feature plot of COL17A1 gene expression in the PDAC atlas dataset (in **e**). Color shows average expression. **i** Dimensional reduction plot (DimPlot) of stromal *versus* ductal (tumor) cells identified by scRNA-Seq in PDAC primary tumors. Populations are color-coded based on the expression of known markers and are visualized using t-SNE. **j** Feature Plot for *L1CAM* expression in multiple cell types identified by scRNA-Seq in PDAC primary tumors. Clusters are color-coded based on *L1CAM* expression and are visualized using t-SNE. **k** Feature Plot for *COL17A1* expression in multiple cell types identified by scRNA-Seq in PDAC primary tumors. Clusters are color-coded based on *L1CAM* expression and are visualized using t-SNE. For all panels, * indicates *p* < 0.05.

To investigate the transcriptional differences between L1_low_ and L1^high^ populations, we FACS sorted metastatic PDAC cells (Supplementary Fig. 1a) followed by mRNA sequencing (RNA-seq). This analysis identified 19,364 differentially expressed genes (DEGs) (Supplementary Table 1). We filtered these genes for the most upregulated (logFC > 2.5) and downregulated (logFC > −2.5) genes with a significance threshold of false discovery rate (FDR < 0.5) and *p*-value < 0.05 in L1_low_ cells compared to L1^high^ cells (Supplementary Fig. 1b). Gene Ontology (GO) analysis revealed an enrichment of genes involved in cell proliferation, adhesion, extracellular matrix (ECM) organization and collagen degradation in L1_low_ cells compared to L1^high^ (Supplementary Fig. 1c). The Kyoto Encyclopedia of Genes and Genomes (KEGG) pathway analysis identified significant enrichment in genes related to cytoskeleton regulation, PPAR signaling, focal adhesion, folate biosynthesis, and axon guidance, suggesting that these pathways may contribute to the altered cellular behavior and aggressiveness of L1_low_ cells (Supplementary Fig. 1d). Gene Set Enrichment Analysis (GSEA) further showed that L1_low_ cells were enriched for genes associated with matrisome composition and collagen formation (Fig. 1a and Supplementary Fig. 1e) compared to L1^high^ cells. Among the most significantly upregulated genes in L1_low_ cells, *COL17A1* stood out (Fig. 1b). Analysis of the TCGA transcriptome dataset demonstrated an inverse correlation between *COL17A1* and *L1CAM* expression in PDAC (Fig. 1c). Notably, *COL17A1* expression was significantly higher in PDAC tissues (*n* = 179) compared to normal pancreatic tissues (NP, *n* = 171) (Fig. 1d). Furthermore, patients with elevated *COL17A1* mRNA levels exhibited poorer prognosis than those with lower levels (Fig. 1e and Supplementary Fig. 2a). Interrogating the single-cell RNA sequencing (scRNA-seq) Atlas database [39], which includes 10 functionally distinct clusters of both tumor and normal samples (Fig. 1f), we observed mutually exclusive expressions patterns of *L1CAM* and *COL17A*1 (Fig. 1f–h). Remarkably, *COL17A1* (Fig. 1h) was highly expressed in the “ductal cell type 2” cluster, composed by malignant epithelial cells. In another scRNA-seq experimental setting where tumors were micro-dissected into epithelial and stromal compartments, we identified 17 distinct cell clusters (Supplementary Fig. 2b–d). We categorized these populations into two groups: “Ductal-like” (epithelial cells) and “Stromal” (fibroblasts, endothelial cells, and immune cells) (Fig. 1i) [35]. In this setting the expression of *L1CAM* and *COL17A1* remained mutually exclusive (Fig. 1j, k). Lastly, FACS analysis confirmed that COL17A1 is predominantly expressed in epithelial tumor cells (L3.6pl and #354) compared to stromal cells (PSC), (Supplementary Fig. 2e). These finding suggest a distinct population of epithelial tumor cells with high *COL17A1* expression in PDAC tumors.

### L1CAM modulation shapes collagen in PDAC tumors

**Fig. 2:**
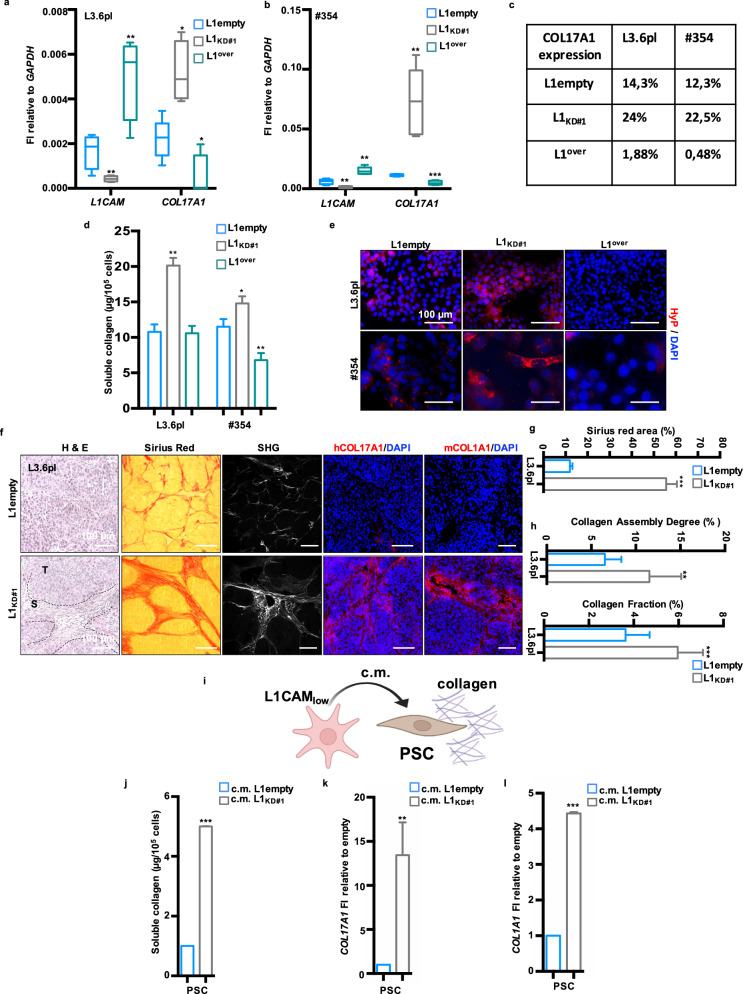
L1 expression influences collagen levels in PDAC. **a** qPCR analysis for *L1* and *COL17A1* expression in L3.6pl cells (L1empty, L1_KD#1_ and L1^over^). Data are normalized to *GAPDH* expression. **b** qPCR analysis for *L1* and *COL17A1* expression in #354 cells (L1empty, L1_KD#1_ and L1^over^). Data are normalized to *GAPDH* expression. **c** Flow cytometry quantification of COL17A1 expression in L3.6pl and #354 cells (L1empty, L1_KD#1_ and L1^over^). **d** Quantification of extracellular collagen by Sircol assay in L3.6pl and #354 (L1empty, L1_KD#1_ and L1^over^) cells. **e** Representative immunofluorescence images for Hydroxyprolin (HyP, red) in L3.6pl and #354 PDAC cells (L1empty, L1_KD#1_ and L1^over^). The nuclei were stained in blue (DAPI). **f** Representative histological sections of xenografts derived from L1empty and L1_KD#2_ L3.6pl cells. Tumor sections were (immuno)stained with Hematoxylin & Eosin (H&E), Sirius Red, Second Armonic Generation (SHG), human COL17A1 and murine COL1A1. The nuclei were stained in blue (DAPI). S stroma, T tumor. **g** Quantification of collagen content in Sirius Red-stained sections. **h** Quantification of Collagen Assembly Degree and Collagen Fraction in SHG images. **i** Schematic representation of conditioned medium (c.m.) experiment. Created with BioRender.com. **j** Quantification of extracellular collagen by Sircol assay in PSC cells grown for 24 h in the presence of L3.6pl (L1empty and L1_KD#1_) c.m. **k** qPCR analysis for *COL17A1* expression in PSC cells grown for 24 h in the presence of L3.6pl (L1empty and L1_KD#1_) c.m. Data are normalized to *GAPDH* expression. *p < 0.05, **p < 0.005, ***p < 0.0005. **l** qPCR analysis for *COL1A1* expression in PSC cells grown for 24 h in the presence of L3.6pl (L1empty and L1_KD#1_) c.m. Data are normalized to *GAPDH* expression. *p < 0.05, **p < 0.005, ***p < 0.0005.

We validated the transcriptomic data through qPCR analysis using both L1CAM-sorted cells (L1_low_ and L1^high^) (Supplementary Fig. 3a, b) and cells engineered for stable downregulation (via lentiviral shRNA, L1_KD_) or upregulation (via lentiviral cDNA expression vector, L1^over^) of *L1CAM* (Fig. 2a, b and Supplementary Fig. 3c–f). Both L1_KD_ and L1_low_ cells exhibited significantly higher levels of *COL17A1* compared to their counterparts, while overexpression of L1CAM showed the opposite trend. The modulation of COL17A1 protein expression based on L1CAM levels was further confirmed by flow cytometry (Fig. 2c and Supplementary Fig. 3g). Next, we employed the Sircol Collagen Assay to measure the in vitro collagen secretion capacity of epithelial cancer cells. This assay revealed that L1_KD_ cells secreted significantly higher quantities of both acid-soluble and pepsin-soluble collagens compared to L1empty and L1^over^ (Fig. 2d and Supplementary Fig. 4a). Hydroxyproline (HyP), a major component of fibrillar collagen crucial for stabilizing collagen structure [40], was analyzed through immunofluorescence (IF) (Fig. 2e) and western blotting (Supplementary Fig. 4b, c), revealing an inverse correlation between L1 and HyP levels. In a previous publication, we demonstrated that L1_low_/_KD_ cells were more aggressive than their high/empty counterparts when subcutaneously injected into the flanks of CD-1 immunocompromised mice [17]. In this study, we histologically analyzed the tumors generated from these two types of cells (Supplementary Fig. 4d) for collagen content. Tumors from L1_low_/L1_KD_ exhibited prominent desmoplasia, as observed by Hematoxylin and Eosin -H&E- staining (Fig. 2f and Supplementary Fig. 4e), along with increased collagen content as indicated by Sirius Red staining (Fig. 2f, g and Supplementary Fig. 4e, f). Using multiphoton microscopy and second harmonic generation (SHG), we assessed structural changes in the extracellular matrix of the tumors. SHG analysis demonstrated an augmented fraction of collagen and an increased assembly degree in tumors generated by L1_low_/L1_KD_ cells compared to their counterparts (Fig. 2f–h and Supplementary Fig. 4e–g). These results corroborate our previous observation that reduced expression of L1 contributes to an increase in collagen content within PDAC tumors. Furthermore, immunofluorescence (Fig. 2f and Supplementary Fig. 4e) confirmed elevated expression of COL17A1 in tumors with low levels of L1. Our findings suggest that while L1_low_ cells contribute to collagen secretion, specifically COL17A1, the abundance of collagen in tumors generated by these cells cannot be solely attributed to epithelial cells. Staining with murine COL1A1 demonstrated significant collagen content in L1_low/KD_-derived tumors (Fig. 2f and Supplementary Fig. 4e). We hypothesized that L1_low_ cells can produce collagen via an autocrine mechanism while also stimulating paracrine secretion in stromal cells (Fig. 2i). This hypothesis was validated in vitro through a soluble collagen assay and qPCR analyses of pancreatic stellate cells (PSCs) treated with conditioned medium (c.m.) from both L1empty and L1_KD_ cells (Fig. 2I, j). Notably, only treatment with c.m. from L1_KD_ cells induced expression of collagen genes *COL17A1* and *COL1A1* in PSCs (Fig. 2k, l and Supplementary Fig. 4h).

### TGF-β1-mediated downregulation of L1CAM enhances COL17A1 and MMP2 levels

**Fig. 3:**
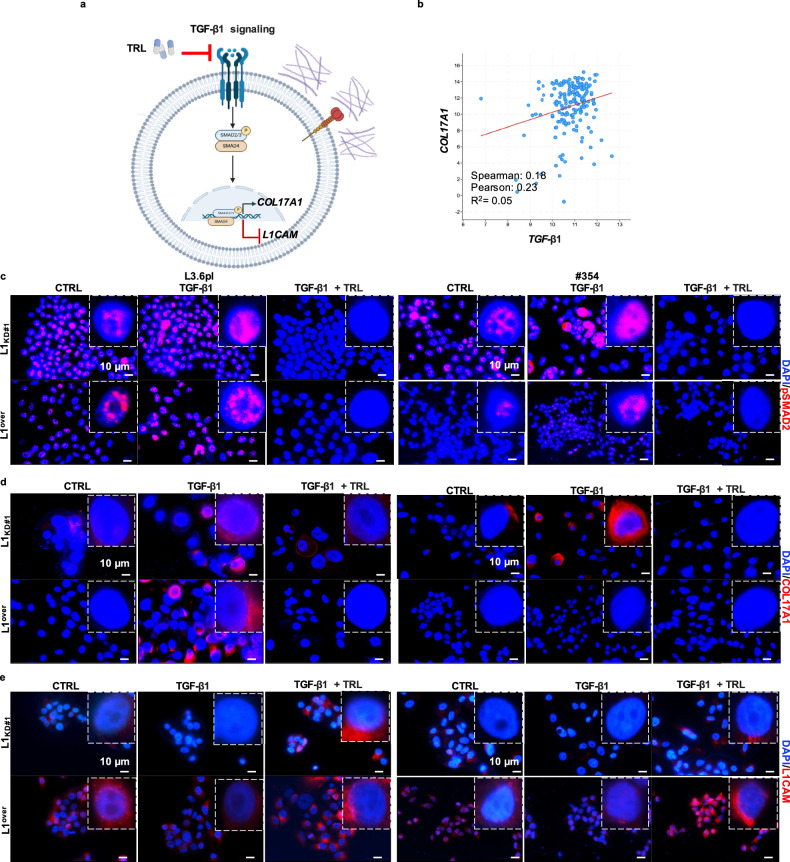
Tranilast-mediated inhibition of TGF-β Signaling reduces COL17A1 levels and enhances L1 expression. **a** Schematic overview of TRL’s mechanism of action in PDAC cells. **b** Positive correlation between *COL17A1* and *TGF-β1* in PDAC samples in the TCGA repository. Created with BioRender.com. **c** Representative immunofluorescence images for pSMAD2 (red) in L3.6pl (left) and #354 (right) PDAC cells (L1_KD#1_ and L1^over^) treated or not with 10 ng/mL of recombinant TGF-β1 in the presence or absence of 120 µM of TRL biweekly for one week. The nuclei were stained in blue (DAPI). **d** Representative immunofluorescence images for COL17A1 (red) in L3.6pl (left) and #354 (right) PDAC cells (L1_KD#1_ and L1^over^) treated or not with 10 ng/mL of recombinant TGF-β1 in the presence or absence of 120 µM of TRL biweekly for one week. The nuclei were stained in blue (DAPI). **e** Representative immunofluorescence images for L1CAM (red) in L3.6pl (left) and #354 (right) PDAC cells (L1_KD#1_ and L1^over^) treated or not with 10 ng/mL of recombinant TGF-β1 in the presence or absence of 120 µM of TRL biweekly for one week. The nuclei were stained in blue (DAPI).

We previously reported that TGF-β1 downregulates L1CAM expression in PDAC, leading to a more aggressive tumor phenotype [17]. Here, we investigated whether the enhanced capacity of cells with low L1CAM levels to upregulate COL17A1 is associated with increased TGF-β1 signaling activity (Fig. 3a). Analysis of the TCGA dataset revealed an inverse correlation between *COL17A1* and *TGF-β1* levels in PDAC (Fig. 3b). To assess the direct involvement of TGF-β1 in modulating COL17A1 levels, we treated L1-empty, L1_KD_, and L1^over^ L3.6pl and #354 cells with recombinant TGF-β1. We observed increased phosphorylation of SMAD2 (pSMAD2), a key mediator of the canonical TGF-β signaling pathway [41], in L1_KD_ cells compared to L1empty and L1^over^ cells across both cell lines (Fig. 3c and Supplementary Fig. 5a, b). Notably, this increase in pSMAD2 levels was accompanied by upregulation of COL17A1 (Fig. 3d) and downregulation of L1CAM (Fig. 3e). To determine whether inhibition of the TGF-β signaling pathway could reverse this phenotype, we tested the effect of Tranilast (TRL), an antifibrotic compound that inhibits Smad2 activation upon stimulation by TGF-β1 [30] and influences collagen matrix deposition [31]. IF analysis demonstrated that TRL effectively reduced pSMAD2 levels induced by TGF-β1 treatment, indicating successful inhibition of TGF-β signaling (Fig. 3c). Furthermore, administration of TRL in combination with TGF-β1 increased L1CAM expression while concurrently decreasing COL17A1 expression (Fig. 3d, e). These results were further confirmed by qPCR and Western Blotting analyses on L3.6pl and #364 wild-type cells, demonstrating that TRL counteracts the effects of TGF-β1 by reducing pSMAD2 and COL17A1 levels and stimulating L1CAM expression (Supplementary Figs. 5c and 6a).

### The increased aggressiveness of L1_low_ cells is counteracted by anti-collagen treatment

**Fig. 4:**
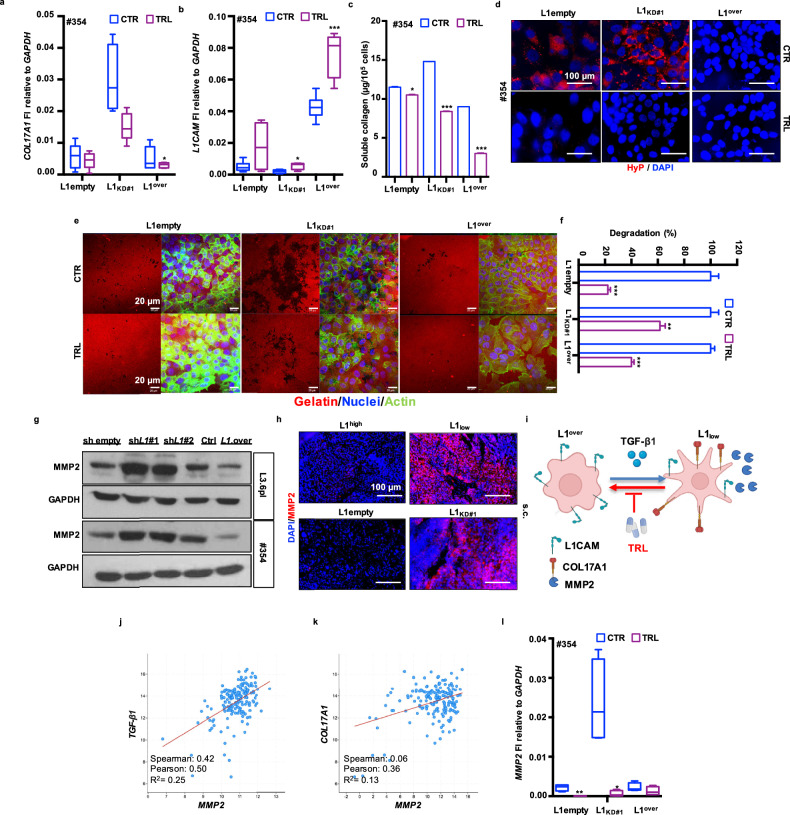
Mitigation of L1_low_-PDAC aggressiveness by Tranilast. **a** qPCR analysis for *COL17A1* expression in #354 cells (L1empty, L1_KD#1_ and L1^over^) treated or not with 120 µM of Tranilast (TRL) biweekly for one week. **b** qPCR analysis for *L1* expression in #354 cells (L1empty, L1_KD#1_ and L1^over^) treated or not with 120 µM of Tranilast (TRL) biweekly for one week. **c** Quantification of extracellular collagen by Sircol assay in #354 (L1empty, L1_KD#1_ and L1^over^) cells treated or not with 120 µM of Tranilast (TRL). **d** Representative immunofluorescence images for Hydroxyprolin (HyP, red) in #354 PDAC cells (L1empty, L1_KD#1_ and L1^over^) treated or not with 120 µM of Tranilast (TRL). The nuclei were stained in blue (DAPI). **e** Representative images of gelatin degradation for L3.6pl (L1empty, L1_KD#1_ and L1^over^) cells treated or not with 120 µM of Tranilast (TRL). Nuclei were stained with Hoechst 33342 (blue), green represents actin (Alexa Fluor™488 Phalloidin) and red illustrates gelatin (Rodhamine). **f** Invasive potential of L3.6pl (L1empty, L1_KD#1_ and L1^over^) cells treated or not with 120 µM of Tranilast (TRL). **g** Western blot analysis for MMP2 in L3.6pl and #354 cells with various L1 levels. Parallel GAPDH immunoblotting was performed. **h** Representative immunofluorescence images for MMP2 (red) in histological sections of xenografts derived from L1^high^, L1_low_, L1empty and L1_KD#1_ L3.6pl cells. The nuclei were stained in blue (DAPI). **i** Schematic representation of Tranilast-Driven TGF-β signaling Inhibition: enhanced L1 expression and suppression of COL17A1 and MMP2 in PDAC. Created with BioRender.com. **j** Positive correlation between *TGF-β1* and *MMP2* in PDAC samples in the TCGA repository. **k** Positive correlation between *COL17A1* and *MMP2* in PDAC samples in the TCGA repository. **l** qPCR analysis for *MMP2* expression in #354 cells (L1empty, L1_KD#1_ and L1^over^) treated or not with 120 µM of Tranilast (TRL) biweekly for one week.

The repurposing of approved antifibrotic drugs that target collagen synthesis and maturation as potential agents against cancer has garnered increased attention [42, 43]. To assess the ability of TRL to inhibit collagen production in PDAC, we performed in vitro treatments using TRL alone on L3.6pl and #354 cells with varying levels of L1 expression. qPCR analysis showed that TRL significantly reduced *COL17A1* expression, particularly in L1_KD_ cells, which exhibited the highest *COL17A1* levels (Fig. 4a, b and Supplementary Fig. 6b). Of note, TRL demonstrated superior effects compared to Halofuginone, a well-known antifibrotic drug (Supplementary Fig. 6c) [44]. Interestingly, the effects of TRL were associated with an increase in L1 expression (Fig. 4a, b and Supplementary Fig. 6b). The soluble collagen assay further confirmed TRL’s effectiveness in diminishing collagen secretion in L1_KD_ cells, with more pronounced effects observed in L1^over^ cells (Fig. 4c). Consistent with these findings, we demonstrated that TRL also reduced collagen secretion from PSC stimulated by c.m. from PDAC cells (Supplementary Fig. 6d). IF analysis revealed that TRL treatment nearly abolished Hyp levels in L1_KD_ cells (L3.6pl) and significantly reduced Hyp levels in #354 cell line (Fig. 4d and Supplementary Fig. 6d). Moreover, TRL suppressed cell aggressiveness by inhibiting migration (Supplementary Fig. 6f, g), and it was found to be more effective than Batimastat (BB-94), a well-known inhibitor of matrix metalloproteinases (MMPs), which play a critical role in cancer migration and tumor spread (Supplementary Fig. 7a, b). Interestingly, TRL also inhibited cellular invasion, demonstrating superior efficacy compared to BB-94 (Fig. 4e, f and Supplementary Fig. 7c–f). RNA-seq analysis suggested that the enhanced aggressiveness of PDAC cells with low L1CAM levels was driven by increased expression of MMPs, particularly *MMP2* (Fig. 1b). We found that *MMP2* expression was significantly higher in PDAC tissues (*n* = 179) compared to normal pancreatic tissues (NP, *n* = 171) (Supplementary Fig. 8a) and its expression correlated with poor prognosis (Supplementary Fig. 8b). qPCR (Supplementary Fig. 8c, d) and western blotting (Fig. 4g) analyses on cells, along with IF on xenografts (Fig. 4h), demonstrated that low L1CAM levels were associated with elevated MMP2 expression compared to their high L1CAM counterparts, confirming the RNA-seq findings. Interestingly, we found that L1_low_ cells also expressed high levels of another metalloproteinase, namely *MMP10*, which is overexpressed in PDAC patients with poorer prognosis (Supplementary Fig. 8e–h). We then assessed whether MMP2 expression was regulated by TGF-β signaling (Fig. 4i). TCGA transcriptome analysis revealed a positive correlation between *TGF-β1* and *MMP2* expression (Fig. 4j), as well as between *COL17A1* and *MMP2* in PDAC tissues (Fig. 4k). Finally, qPCR analysis showed a reduction in *MMP2* expression following TRL treatment (Fig. 4l).

### TRL suppresses liver metastasis driven by L1_low_ cells

**Fig. 5:**
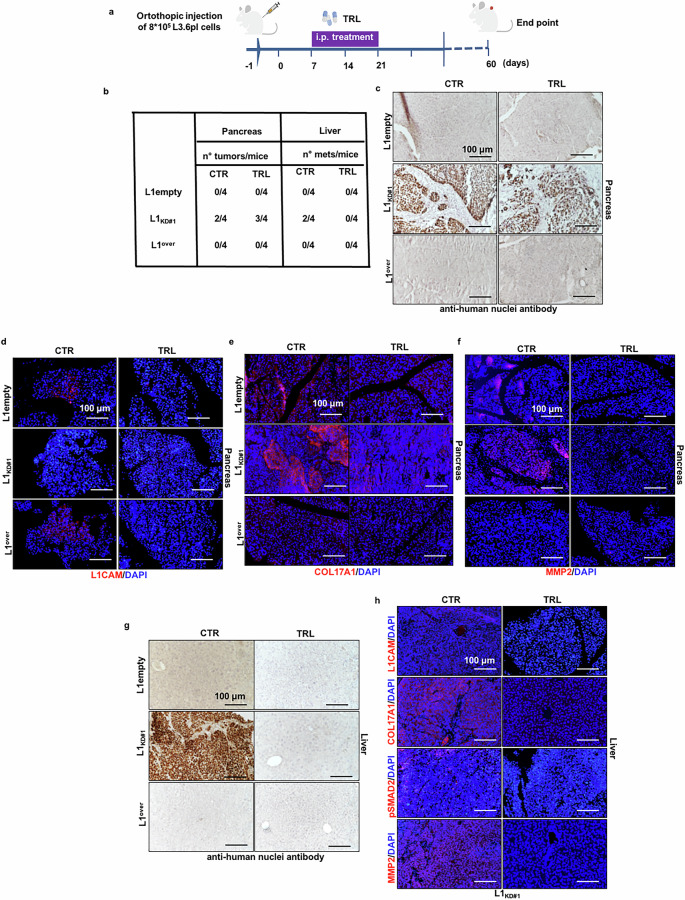
Tranilast attenuates liver metastasis driven by L1_low_ PDAC cells. **a** Schematic representation of orthotopic injection of L3.6pl (L1empty, L1_KD#1_ and L1^over^) cells into the pancreas of 6-week-old nude CD1 male mice. Mice were treated biweekly for three weeks with TRL (50 mg/kg of mice). Created with BioRender.com. **b** Number of tumors and liver metastasis generated from mice orthotopically injected with L3.6pl cells, treated or not with TRL (50 mg/kg of mice). **c** Representative histological sections of pancreas derived from mice injected with L3.6pl cells, treated or not with TRL. Tumor sections were (immuno)stained for human nuclei. **d** Representative histological sections of pancreas derived from mice injected with L3.6pl cells, treated or not with TRL. Tumor sections were (immuno)stained for L1CAM. The nuclei were stained in blue (DAPI). **e** Representative histological sections of pancreas derived from mice injected with L3.6pl cells, treated or not with TRL. Tumor sections were (immuno)stained for human COL17A1. The nuclei were stained in blue (DAPI). **f** Representative histological sections of pancreas derived from mice injected with L3.6pl cells, treated or not with TRL. Tumor sections were (immuno)stained for MMP2. The nuclei were stained in blue (DAPI). **g** Representative histological sections of livers derived from mice injected with L3.6pl cells, treated or not with TRL. Tumor sections were (immuno)stained for human nuclei. **h** Representative histological sections of pancreas derived from mice injected with L3.6pl cells, treated or not with TRL. Tumor sections were (immuno)stained for L1CAM, COL17A1, pSMAD2, MMP2, respectively. The nuclei were stained in blue (DAPI).

To assess the capability of TRL in inhibiting tumor growth and cellular migration in vivo (i.e., the formation of metastases in distant organs), we injected L1empty, L1_KD_, and L1^over^ cells orthotopically into the pancreas of CD-1 immunocompromised mice, followed by intraperitoneal administration of TRL according to the schedule outlined in Fig. 5a. Mice were sacrificed 60 days post-injection. We demonstrated that only L1_KD_ cells were capable of initiating tumors upon injection into the pancreas, followed by the development of liver metastases (Fig. 5b). Histological examination using an anti-human nuclei antibody (Fig. 5c) and H&E staining (Supplementary Fig. 8i) confirmed the presence of primary tumors and liver metastases. We didn’t detect L1CAM expression in the pancreas of mice injected with L1_KD_ cells (Fig. 5d), while tumors showed positive staining for COL17A1 and MMP2 (Fig. 5e, f). Notably, although treatment with TRL had no effect on the inhibition of primary tumor formation in the pancreas (Fig. 5b), it significantly reduced the expression of COL17A1 and MMP2 (Fig. 5e, f). In the liver, TRL treatment markedly decreased the formation of metastases exclusively formed by L1_KD_ cells (Fig. 5b, g), which was accompanied by an increase in L1CAM levels and a reduction in COL17A1, pSMAD2 and MMP2 levels (Fig. 5h). Given that it is unlikely that only L1_KD_ cells are secreting large amounts of collagen, we hypothesized that the effects could also be non-cell autonomous, potentially involving recruitment of murine stromal cells. IHC using a murine-specific marker for COL1A1 revealed strong positivity for this marker (Supplementary Fig. 8j). However, TRL treatment did not interfere with COL1A1 expression in primary tumors (Supplementary Fig. 8j). In contrast, we observed a significant reduction in COL1A1 expression following TRL treatment at the metastatic site (Supplementary Fig. 8k).

### TRL synergizes with gemcitabine to reduce tumor fibrosis

**Fig. 6:**
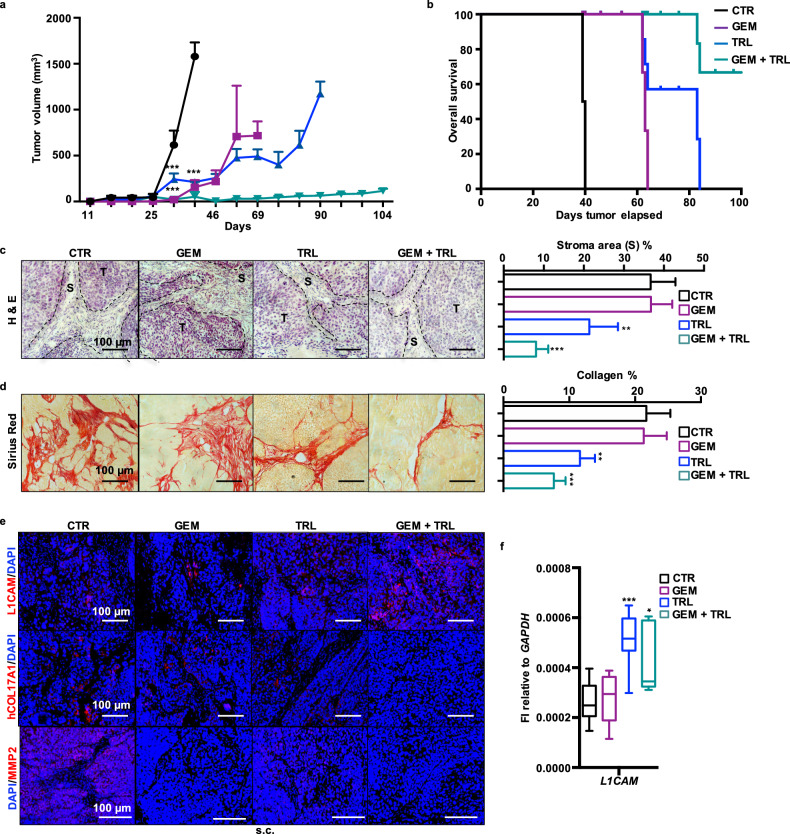
Tranilast and Gemcitabine synergistically affect tumor growth and fibrosis. **a** Tumor volume of #354 cells subcutaneously xenografted, treated or not with Gem (100 mg/kg of mice) and/or TRL (50 mg/kg of mice) over 104 days. **b** Kaplan–Meier curve of #354 cells subcutaneously xenografted, treated or not with Gem (100 mg/kg of mice) and/or TRL (50 mg/kg of mice). **c** Left: Representative histological sections of xenografts derived from #354 cells. Tumor sections were stained with Hematoxylin & Eosin (H&E). S stroma; T tumor. Right: quantification of stroma content on H&E-stained sections. **d** Left: Representative histological sections of xenografts derived from #354 cells. Tumor sections were stained with Sirius Red. Right: quantification of collagen content on Sirius Red-stained sections. **e** Representative histological sections of xenografts derived from #354 cells. Tumor sections were (immuno)stained for human L1CAM, COL17A1 and MMP2. The nuclei were stained in blue (DAPI). **f** qPCR analysis for *L1CAM* expression in #354-derived tumors. Data are normalized to *GAPDH* expression. *p < 0.05, **p < 0.005, ***p < 0.0005.

To evaluate the capacity of TRL to synergize with Gemcitabine (GEM) we conducted an in vivo experiment involving the subcutaneous injection of #354 cells into nude mice (Fig. 6a). Both GEM and TRL individually demonstrated the ability to decrease tumor volume without any observed toxicity in the animals compared to vehicle-treated mice (CTR). However, this effect was significantly enhanced and maintained for up to 104 days in the co-treatment group receiving GEM plus TRL (green line, Fig. 6a, b). H&E staining revealed a significant reduction in tumor stroma (S) with dual treatment (Fig. 6c). Additionally, Sirius Red staining confirmed a substantial decrease in collagen content (Fig. 6d). Interestingly, TRL treatment diminished the expression of both COL17A1 and murine COL1A1 (Fig. 6e and Supplementary Fig. 8l), with this reduction being particularly pronounced in the dual treatment group (Fig. 6e). Only untreated tumors exhibited high levels of MMP2, which were significantly reduced by both individual treatments and the combined therapy. Furthermore, qPCR analyses performed on RNA extracted from the tumors showed heightened expression of L1CAM in mice treated with TRL (Fig. 6f). These findings were further supported by IF analysis on tumor sections, confirming the upregulation of L1CAM following TRL treatment, with an even more pronounced effect observed in the co-treatment group.

## Discussion

The highly fibrotic microenvironment of pancreatic tumors has garnered significant attention in the context of drug-targeting approaches and molecular strategies [45, 46]. However, the effectiveness of many interventions has been limited [5]. For example, halofuginone and pirfenidone, known antifibrotic agents, have shown promise in modulating collagen matrix architecture in various cancer models, including PDAC, while Losartan, an FDA-approved anti-hypertensive drug, has demonstrated antifibrotic effects by suppressing active TGF-β1 levels [44, 47]. Similarly, tamoxifen, a selective estrogen receptor modulator, has been implicated in reducing hypoxia-inducible factor-1 alpha (HIF-1α) levels and altering collagen deposition in PDAC models [48]. While antifibrotic drugs hold potential for promoting tumor tissue remodeling and enhancing drug delivery, their long-term effects on tumor spread remain a critical concern [38]. In our previous work, we demonstrated that TGF-β1 negatively regulates L1 cell adhesion molecule (L1CAM) expression through canonical TGF-β-Smad2/3 signaling, leading to a more invasive PDAC phenotype [11]. Our current study advances the understanding of PDAC pathophysiology by elucidating the role of L1CAM in the dynamics of collagen deposition within the tumor microenvironment. Collagen is well recognized for its impact on cancer-associated fibrosis, tissue stiffness, and metastasis; in the context of PDAC—a malignancy notorious for its desmoplastic reaction—the modulation of collagen plays a central role in disease progression and treatment resistance [43, 49, 50]. Remarkably, our findings challenge the preconceived notion that collagen regulation is predominantly a function of stromal cells [51]. Instead, we propose that specific tumor epithelial subsets, particularly those with low L1CAM expression (L1_low_), can actively influence collagen dynamics. Transcriptomic analysis of PDAC cells based on L1 expression levels unveiled a surprising shift: L1_low_ cells were identified as having upregulated collagen 17A1 (*COL17A1*), suggesting their unexpected role in collagen secretion. *COL17A1*, which encodes for collagen XVII, has been linked to pancreatic cancer; studies have shown that high levels of COL17A1 expression are associated with a poor prognosis for pancreatic cancer patients [39]. This characteristic of L1_low_ cells extends beyond isolated in vitro finding to enhanced migratory abilities, aligning with broader capabilities required for tumor invasion and metastasis [11]. Specifically, L1_low_ cells upregulate matrix metalloproteinases MMP2 and MMP10, enzymes that degrade ECM components to facilitate tumor dissemination. In vivo, the L1_low_ phenotype correlates with robust collagen deposition, resistance to gemcitabine, and increased tendency liver metastases- a process likely driven by MMP2/MMP10 – mediated ECM remodeling. These findings underscore the interplay between L1 expression, TME composition, and the aggressive metastatic behavior of PDAC. The intervention using Tranilast- traditionally recognized as an anti-fibrotic agent [34]- provided significant insights into potential therapeutic strategies. Tranilast treatment not only attenuated tumor volume and collagen deposition but also appeared to promote L1CAM expression. Notably, Tranilast outperformed Halofuginone in collagen reduction and Batimastat in inhibiting migration, suggesting its dual utility in mitigating fibrosis and tumor spread. Mechanistically, we found that high levels of TGF-β1 in the microenvironment reduce L1 expression on PDAC cells undergoing phenotypic transformation, rendering them more aggressive while enhancing their ability to produce and secrete COL17A1 and MMP2 - both positively associated with poor prognosis. Targeting COL17A1 via upstream TGF-β1 blockade using Tranilast or potentially other compounds such as Galunisertib [52] or Vactosertib may reduce tumor progression and cellular aggressiveness. Clinically, Tranilast is well-tolerated at doses up to 600 mg daily, making it an attractive candidate for managing PDAC fibrosis. Patients with low L1CAM expression may benefit from combining Tranilast with Gemcitabine. However, TGF-β1 inhibition carries risks: TGF-β1 also modulates immune responses, and its suppression could compromise immune surveillance or promote metastasis in some patients. PDAC heterogeneity further complicates this approach, as compensatory pathways may arise post-TGF-β1 blockade. Combination therapies with chemotherapy or immunotherapy warrant exploration, though clinical studies are needed to optimize dosing, safety, and patient stratification. This study advances our understanding of PDAC pathophysiology in two key ways. First, it identifies L1CAM as a regulator of collagen secretion, redefining epithelial contributions to the TME and highlighting potential biomarkers for disease severity and treatment response. Second, it positions Tranilast as a promising adjunct to conventional chemotherapy, with the capacity to remodel the TME and target metastatic mechanisms.

## Supplementary information

Supplementary Data

western blotting

Supplementary Table 1

## References

[1] Bray F, Ferlay J, Soerjomataram I, Siegel RL, Torre LA, Jemal A. Global cancer statistics 2018: GLOBOCAN estimates of incidence and mortality worldwide for 36 cancers in 185 countries. CA Cancer J Clinicians. 2018;68:394–424. 10.3322/caac.21492 30207593

[2] Rawla P, Sunkara T, Gaduputi V. Epidemiology of pancreatic cancer: global trends, etiology and risk factors. World J Oncol. 2019;10:10–27. 30834048 10.14740/wjon1166 PMC6396775

[3] Van den Broeck A, Vankelecom H, Van Eijsden R, Govaere O, Topal B. Molecular markers associated with outcome and metastasis in human pancreatic cancer. J Exp Clin Cancer Res. 2012;31:68. 22925330 10.1186/1756-9966-31-68 PMC3511800

[4] Gresham GK, Wells GA, Gill S, Cameron C, Jonker DJ. Chemotherapy regimens for advanced pancreatic cancer: a systematic review and network meta-analysis. BMC Cancer. 2014;14:471. 24972449 10.1186/1471-2407-14-471 PMC4097092

[5] Shinkawa T, Ohuchida K, Mochida Y, Sakihama K, Iwamoto C, Abe T, et al. Subtypes in pancreatic ductal adenocarcinoma based on niche factor dependency show distinct drug treatment responses. J Exp Clin Cancer Res. 2022;41:89. 35272688 10.1186/s13046-022-02301-9 PMC8908673

[6] Hosein AN, Brekken RA, Maitra A. Pancreatic cancer stroma: an update on therapeutic targeting strategies. Nat Rev Gastroenterol Hepatol. 2020;17:487–505. 32393771 10.1038/s41575-020-0300-1 PMC8284850

[7] Ho WJ, Jaffee EM, Zheng L. The tumour microenvironment in pancreatic cancer — clinical challenges and opportunities. Nat Rev Clin Oncol. 2020;17:527–40. 32398706 10.1038/s41571-020-0363-5 PMC7442729

[8] Cave DD, Buonaiuto S, Sainz B, Fantuz M, Mangini M, Carrer A, et al. LAMC2 marks a tumor-initiating cell population with an aggressive signature in pancreatic cancer. J Exp Clin Cancer Res. 2022;41:315. 36289544 10.1186/s13046-022-02516-w PMC9609288

[9] Öhlund D, Handly-Santana A, Biffi G, Elyada E, Almeida AS, Ponz-Sarvise M, et al. Distinct populations of inflammatory fibroblasts and myofibroblasts in pancreatic cancer. J Exp Med. 2017;214:579–96. 28232471 10.1084/jem.20162024 PMC5339682

[10] Puls TJ, Tan X, Whittington CF, Voytik-Harbin SL. 3D collagen fibrillar microstructure guides pancreatic cancer cell phenotype and serves as a critical design parameter for phenotypic models of EMT. PLoS One. 2017;12:e0188870. 29190794 10.1371/journal.pone.0188870 PMC5708668

[11] Elia I, Rossi M, Stegen S, Broekaert D, Doglioni G, Van Gorsel M, et al. Breast cancer cells rely on environmental pyruvate to shape the metastatic niche. Nature. 2019;568:117–21. 30814728 10.1038/s41586-019-0977-x PMC6451642

[12] D’Aniello C, Cermola F, Palamidessi A, Wanderlingh LG, Gagliardi M, Migliaccio A, et al. Collagen prolyl hydroxylation–dependent metabolic perturbation governs epigenetic remodeling and mesenchymal transition in pluripotent and cancer cells. Cancer Res. 2019;79:3235–50. 31061065 10.1158/0008-5472.CAN-18-2070

[13] Ippolito L, Duatti A, Iozzo M, Comito G, Pardella E, Lorito N et al. Lactate supports cell-autonomous ECM production to sustain metastatic behavior in prostate cancer. EMBO Rep. 2024. 10.1038/s44319-024-00180-z. 10.1038/s44319-024-00180-z PMC11315984 38907027

[14] Qiu B, Wei F, Sun X, Wang X, Duan B, Shi C et al. Measurement of hydroxyproline in collagen with three different methods. Mol Med Rep. 2014;10:1157–63. 10.3892/mmr.2014.2267 24858249

[15] Tian C, Huang Y, Clauser KR, Rickelt S, Lau AN, Carr SA, et al. Suppression of pancreatic ductal adenocarcinoma growth and metastasis by fibrillar collagens produced selectively by tumor cells. Nat Commun. 2021;12:2328. 33879793 10.1038/s41467-021-22490-9 PMC8058088

[16] Olivares O, Mayers JR, Gouirand V, Torrence ME, Gicquel T, Borge L, et al. Collagen-derived proline promotes pancreatic ductal adenocarcinoma cell survival under nutrient limited conditions. Nat Commun. 2017;8:16031. 28685754 10.1038/ncomms16031 PMC5504351

[17] Cave DD, Di Guida M, Costa V, Sevillano M, Ferrante L, Heeschen C, et al. TGF-β1 secreted by pancreatic stellate cells promotes stemness and tumourigenicity in pancreatic cancer cells through L1CAM downregulation. Oncogene. 2020;39:4271–85. 32291413 10.1038/s41388-020-1289-1 PMC7239770

[18] Tang D-Y, Yu Y, Zhao X-J, Schachner M, Zhao W-J. Single chain fragment variable antibodies developed by using as target the 3rd fibronectin type III homologous repeat fragment of human neural cell adhesion molecule L1 promote cell migration and neuritogenesis. Exp Cell Res. 2015;330:336–45. 25447207 10.1016/j.yexcr.2014.10.021

[19] Grumet M. The L1CAM extracellular region a multi-domain protein with modular and cooperative binding modes. Front Biosci. 2003;8:s1210–1225. 12957823 10.2741/1108

[20] Ganesh, K. L1CAM defines the regenerative origin of metastasis-initiating cells in colorectal cancer. Nat Cancer. 2020;1:28–45. 10.1038/s43018-019-0006-x PMC7351134 32656539

[21] Cave DD, Hernando-Momblona X, Sevillano M, Minchiotti G, Lonardo E. Nodal-induced L1CAM/CXCR4 subpopulation sustains tumor growth and metastasis in colorectal cancer derived organoids. Theranostics. 2021;11:5686–99. 33897875 10.7150/thno.54027 PMC8058729

[22] Schröder C, Schumacher U, Fogel M, Feuerhake F, Müller V, Wirtz RM, et al. Milde-Langosch. Expression and prognostic value of L1-CAM in breast cancer. Oncol Rep. 2009;22:1109–17. 19787228 10.3892/or_00000543

[23] Giordano M, Decio A, Battistini C, Baronio M, Bianchi F, Villa A, et al. L1CAM promotes ovarian cancer stemness and tumor initiation via FGFR1/SRC/STAT3 signaling. J Exp Clin Cancer Res. 2021;40:319. 34645505 10.1186/s13046-021-02117-z PMC8513260

[24] Altevogt P, Doberstein K, Fogel M. L1CAM in human cancer. Int J Cancer. 2016;138:1565–76. 26111503 10.1002/ijc.29658

[25] Kaifi JT, Heidtmann S, Schurr PG, Reichelt U, Mann O, Yekebas EF et al. Absence of L1 in pancreatic masses distinguishes adenocarcinomas from poorly differentiated neuroendocrine carcinomas. Anticancer Res. 2006;26:1167–70. 16619519

[26] Tsutsumi S, Morohashi S, Kudo Y, Akasaka H, Ogasawara H, Ono M, et al. L1 Cell adhesion molecule (L1CAM) expression at the cancer invasive front is a novel prognostic marker of pancreatic ductal adenocarcinoma: L1CAM Expression in Pancreatic Cancer. J Surg Oncol. 2011;103:669–73. 21360711 10.1002/jso.21880

[27] Ben Q-W, Wang J-C, Liu J, Zhu Y, Yuan F, Yao W-Y, et al. Positive expression of L1-CAM is associated with perineural invasion and poor outcome in pancreatic ductal adenocarcinoma. Ann Surg Oncol. 2010;17:2213–21. 20162456 10.1245/s10434-010-0955-x

[28] Sebens Müerköster. Elevated L1CAM expression in precursor lesions and primary and metastastic tissues of pancreatic ductal adenocarcinoma. Oncol Rep. 2010;24. 10.3892/or.2010.909. 10.3892/or.2010.909 20811670

[29] Osman S, Raza A, Al-Zaidan L, Inchakalody VP, Merhi M, Prabhu KS, et al. Anti-cancer effects of Tranilast: an update. Biomed Pharmacother. 2021;141:111844. 34174504 10.1016/j.biopha.2021.111844

[30] Takahashi K, Nishikawa S, Miyata R, Noguchi M, Ishikawa H, Yutaka Y, et al. Tranilast inhibits TGF-beta-induced EMT and invasion/metastasis via the suppression of smad4 in lung cancer cell lines. Ann Oncol. 2018;29:viii10. 10.21873/anticanres.14311 32487624

[31] Yamada H, Tajima S, Nishikawa T, Murad S, Pinnell SR. Tranilast, a selective inhibitor of collagen synthesis in human skin fibroblasts. J Biochem. 1994;116:892–7. 7533764 10.1093/oxfordjournals.jbchem.a124612

[32] Lonardo E, Hermann PC, Mueller M-T, Huber S, Balic A, Miranda-Lorenzo I, et al. Nodal/activin signaling drives self-renewal and tumorigenicity of pancreatic cancer stem cells and provides a target for combined drug therapy. Cell Stem Cell. 2011;9:433–46. 22056140 10.1016/j.stem.2011.10.001

[33] Varone A, Amoruso C, Monti M, Patheja M, Greco A, Auletta L, et al. The phosphatase Shp1 interacts with and dephosphorylates cortactin to inhibit invadopodia function. Cell Commun Signal. 2021;19:64. 34088320 10.1186/s12964-021-00747-6 PMC8176763

[34] Hao Y, Stuart T, Kowalski MH, Choudhary S, Hoffman P, Hartman A, et al. Dictionary learning for integrative, multimodal and scalable single-cell analysis. Nat Biotechnol. 2024;42:293–304. 37231261 10.1038/s41587-023-01767-y PMC10928517

[35] Lin W, Noel P, Borazanci EH, Lee J, Amini A, Han IW, et al. Single-cell transcriptome analysis of tumor and stromal compartments of pancreatic ductal adenocarcinoma primary tumors and metastatic lesions. Genome Med. 2020;12:80. 32988401 10.1186/s13073-020-00776-9 PMC7523332

[36] Xu Q, Chen S, Hu Y, Huang W. Single-cell RNA transcriptome reveals the intra-tumoral heterogeneity and regulators underlying tumor progression in metastatic pancreatic ductal adenocarcinoma. Cell Death Discov. 2021;7:331. 34732701 10.1038/s41420-021-00663-1 PMC8566471

[37] Peng J, Sun B-F, Chen C-Y, Zhou J-Y, Chen Y-S, Chen H, et al. Single-cell RNA-seq highlights intra-tumoral heterogeneity and malignant progression in pancreatic ductal adenocarcinoma. Cell Res. 2019;29:725–38. 31273297 10.1038/s41422-019-0195-y PMC6796938

[38] Xie P, Gao M, Wang C, Zhang J, Noel P, Yang C, et al. SuperCT: a supervised-learning framework for enhanced characterization of single-cell transcriptomic profiles. Nucleic Acids Res. 2019;47:e48–e48. 30799483 10.1093/nar/gkz116 PMC6486558

[39] Chijimatsu R, Kobayashi S, Takeda Y, Kitakaze M, Tatekawa S, Arao Y, et al. Establishment of a reference single-cell RNA sequencing dataset for human pancreatic adenocarcinoma. iScience. 2022;25:104659. 35847558 10.1016/j.isci.2022.104659 PMC9283889

[40] Xu S, Gu M, Wu K, Li G. Unraveling the role of hydroxyproline in maintaining the thermal stability of the collagen triple helix structure using simulation. J Phys Chem B. 2019;123:7754–63. 31418574 10.1021/acs.jpcb.9b05006

[41] Yokobori T, Nishiyama M. TGF-β signaling in gastrointestinal cancers: progress in basic and clinical research. JCM. 2017;6:11. 28106769 10.3390/jcm6010011 PMC5294964

[42] Xia Y, Sun M, Huang H, Jin W-L. Drug repurposing for cancer therapy. Sig Transduct Target Ther. 2024;9:92. 10.1038/s41392-024-01808-1 PMC11026526 38637540

[43] Ibello E, Saracino F, Delle Cave D, Buonaiuto S, Amoroso F, Andolfi G, et al. Three-dimensional environment sensitizes pancreatic cancer cells to the anti-proliferative effect of budesonide by reprogramming energy metabolism. J Exp Clin Cancer Res. 2024;43:165. 38877560 10.1186/s13046-024-03072-1 PMC11177459

[44] Zion O, Genin O, Kawada N, Yoshizato K, Roffe S, Nagler A, et al. Inhibition of transforming growth factor β signaling by halofuginone as a modality for pancreas fibrosis prevention. Pancreas. 2009;38:427–35. 19188864 10.1097/MPA.0b013e3181967670

[45] Alemanno F, Cavo M, Delle Cave D, Fachechi A, Rizzo R, D’Amone E, et al. Quantifying heterogeneity to drug response in cancer–stroma kinetics. Proc Natl Acad Sci USA. 2023;120:e2122352120. 36897966 10.1073/pnas.2122352120 PMC10089157

[46] Adamopoulos C, Cave DD, Papavassiliou AG. Inhibition of the RAF/MEK/ERK signaling cascade in pancreatic cancer: recent advances and future perspectives. IJMS. 2024;25:1631. 38338909 10.3390/ijms25031631 PMC10855714

[47] Boucher Y, Posada JM, Subudhi S, Kumar AS, Rosario SR, Gu L, et al. Addition of Losartan to FOLFIRINOX and chemoradiation reduces immunosuppression-associated genes, tregs, and FOXP3+ cancer cells in locally advanced pancreatic cancer. Clin Cancer Res. 2023;29:1605–19. 36749873 10.1158/1078-0432.CCR-22-1630 PMC10106451

[48] Pein M, Oskarsson T. Tamoxifen calms down the distressed PDAC stroma. EMBO Rep. 2019;20:e47334. 30538119 10.15252/embr.201847334 PMC6322357

[49] Boyd LNC, Andini KD, Peters GJ, Kazemier G, Giovannetti E. Heterogeneity and plasticity of cancer-associated fibroblasts in the pancreatic tumor microenvironment. Semin Cancer Biol. 2022;82:184–96. 33737108 10.1016/j.semcancer.2021.03.006

[50] Kashiwagi R, Funayama R, Aoki S, Matsui A, Klein S, Sato Y, et al. Collagen XVII regulates tumor growth in pancreatic cancer through interaction with the tumor microenvironment. Cancer Sci. 2023;114:4286–98. 37688308 10.1111/cas.15952 PMC10637054

[51] Ogawa Y, Masugi Y, Abe T, Yamazaki K, Ueno A, Fujii-Nishimura Y, et al. Three distinct stroma types in human pancreatic cancer identified by image analysis of fibroblast subpopulations and collagen. Clin Cancer Res. 2021;27:107–19. 33046515 10.1158/1078-0432.CCR-20-2298

[52] Delle Cave D, Mangini M, Tramontano C, De Stefano L, Corona M, Rea I, et al. Hybrid biosilica nanoparticles for in-vivo targeted inhibition of colorectal cancer growth and label-free imaging. IJN. 2024;19:12079–98. 39583322 10.2147/IJN.S480168 PMC11585298

