# Supplementary material for: TGF-β1-mediated downregulation of L1CAM in pancreatic ductal adenocarcinoma drives upregulation of collagen 17A1 and MMP2, facilitating tumor invasiveness and metastasis
Source: Cell Death Dis. 2025 Aug 6;16(1):592. doi: 10.1038/s41419-025-07859-8 (PMC12328658; doi:10.1038/s41419-025-07859-8)

Supplementary Figure Legend:

Supplementary Fig. 1:

a. Representative flow cytometry for L1 in L3.6pl and 354 cells. All cytometry gates were established based on isotype controls**. b.** Heatmap showing the differentially expressed genes between L1low and L1high cells based on RNA-seq analysis. **c.** Gene ontology (GO) enrichment analysis showing the most upregulated genes and associated pathways in $L1_{low}$ respect to $L1^{high}$ cells based on RNA-seq data. **d.** KEGG pathway enrichment analysis showing the most upregulated genes and associated pathways in L1low and L1high cells based on RNA-seq data. **e**. Enrichment plot for $L1_{low}$ versus $L1^{high}$ FACS-sorted L3.6pl cells.

Supplementary Fig. 2:

a. Kaplan–Meier curves showing overall survival of PDAC patients, stratified according to the median value of *COL17A1* expression. **b.** Dimensional reduction plot (DimPlot) of multiple cell types identified in PDAC primary tumors by single-cell RNA sequencing (scRNA-Seq). The clusters are color-coded based on cell types identified using known cell type-specific markers and are visualized using t-Distributed Stochastic Neighbor embedding (t-SNE). **c.** Bar plot displaying the supervised prediction of cell types contained in cluster 7 of the GSE154778 dataset, obtained via the SuperCT suite (Xie et al., Nucleic Acids Research, 2019). **d.** Violin plot of L1CAM normalized expression values grouped by Seurat annotated cell cluster. **e.** Representative flow cytometry for COL17A1 in PSC, L3.6pl and #354 wild type cells. All cytometry gates were established based on isotype controls.

Supplementary Fig. 3:

a. qPCR analysis for *L1* and *COL17A1* expression in L3.6pl cells ($L1_{low}$ and $L1^{high}$). Data are normalized to *GAPDH* expression. **b.** qPCR analysis for *L1* and *COL17A1* expression in #354 cells ($L1_{low}$ and $L1^{high}$). Data are normalized to *GAPDH* expression. **c.** qPCR analysis for *L1* and *COL17A1* expression in L3.6pl cells (L1empty and $L1_{KD\#2}$). Data are normalized to *GAPDH* expression. **d.** qPCR analysis for *L1* and *COL17A1* expression in #354 cells (L1empty and $L1_{KD\#2}$). Data are normalized to *GAPDH* expression. **e.** Representative flow cytometry for L1 in L3.6pl (L1empty, $L1_{KD\#1}$, $L1_{KD\#2}$, WT and $L1^{over}$) cells. All cytometry gates were established based on isotype controls. **f.** Representative flow cytometry for L1 in #354 (L1empty, $L1_{KD\#1}$, $L1_{KD\#2}$, WT and $L1^{over}$) cells. All cytometry gates were established based on isotype controls. **g.** Representative flow cytometry for COL17A1 in L3.6pl and #354 (L1empty, $L1_{KD\#1}$, WT and $L1^{over}$) cells. All cytometry gates were established based on isotype controls. $^{*}p < 0.05$, $^{**}p < 0.005$

Supplementary Fig. 4:

a. Quantification of extracellular collagen by Sircol assay on L3.6pl and #354 (L1empty and $L1_{KD\#2}$) cells. **b.** Western blot analysis of HyP in L3.6pl and #354 cells (L1empty, $L1_{KD\#1}$, $L1_{KD\#2}$, WT and $L1^{over}$). Parallel GAPDH immunoblotting was performed. **c**. Quantification of HyP levels calculated by densitometric analysis.

d. Representative histological sections of xenografts derived from L1 sorted cells ($L1_{low}$ and $L1^{high}$). Tumor sections were (immuno)stained for human L1CAM. **e.** Representative histological sections of xenografts derived from L1 sorted cells ($L1_{low}$ and $L1^{high}$) and $L1_{KD\#2}$. Tumor sections were (immuno)stained for Hematoxylin & Eosin (H&E), Sirius Red, Second Armonic Generation (SHG), human COL17A1 and murine COL1A1. The nuclei were stained in blue (DAPI). S: stroma; T: tumor. **f.** Quantification of collagen content on Sirius Red stained sections. **g.** Quantification of Collagen Assembly Degree (left) and Collagen Fraction (right) on SHG images. **h.** qPCR analysis for *COL17A1* expression in PSC cells grown for 24 hours in the presence of L3.6pl (L1empty and $L1_{KD\#2}$) c.m. Data are normalized to *GAPDH* expression. *p < 0.05, **p < 0.005, ***p < 0.0005.

Supplementary Fig. 5:

a. Representative immunofluorescence images for pSMAD2 (red) in L3.6pl PDAC cells ($L1_{KD\#1}$ and $L1^{over}$) treated or not with 10 ng/mL of recombinant TGF-β1 in the presence or absence of 120 μM of TRL biweekly for one week. The nuclei were stained in blue (DAPI). **b.** Representative immunofluorescence images for pSMAD2 (red) in #354 PDAC cells ($L1_{KD\#1}$ and $L1^{over}$) treated or not with 10 ng/mL of recombinant TGF-β1 in the presence or absence of 120 μM of TRL biweekly for one week. The nuclei were stained in blue (DAPI). **c.** qPCR analysis for L1 and COL17A1 expression in L3.6pl (left) and #354 (right) wild type cells with 10 ng/mL of recombinant TGF-β1 in the presence or absence of 120 μM of TRL biweekly for one week. *p < 0.05, **p < 0.005, ***p < 0.0005.

Supplementary Fig. 6:

a. Western blot analysis of pSMAD2 and SMAD2 in #354 cells treated or not with 10 ng/mL of TGF-β1 in the presence or absence of 120 μM of Tranilast (TRL). **b.** qPCR analysis for *L1* (left) and *COL17A1* (right) expression in #L3.6pl cells (L1empty, $L1_{KD\#1}$ and $L1^{over}$) treated or not with 120 μM of Tranilast (TRL) biweekly for one week. **c.** qPCR analysis for *COL1A1*, *COL5A2*, *COL7A1*, *COL12A1* and *COL17A1* expression in #L3.6pl cells ($L1_{KD\#1}$ and $L1^{over}$) treated or not with 50 nM of Halofuginone (Halo) biweekly for one week. **d.** Quantification of extracellular collagen by Sircol assay on PSC cells treated with c.m. of L3.6pl ($L1_{KD\#1}$ and $L1^{over}$) cells treated or not with 120 μM of Tranilast (TRL). **e.** Representative immunofluorescence images for Hydroxyprolin (HyP, red) in L3.6pl PDAC cells (L1empty, $L1_{KD\#1}$ and $L1^{over}$) treated or not with 120 μM of Tranilast (TRL). The nuclei were stained in blue (DAPI). **f.** Representative images of migration capacity (boyden chamber assay) for L3.6pl (L1empty, $L1_{KD\#1}$ and $L1^{over}$) cells treated or not with 120 μM of Tranilast (TRL). **g.** Migratory potential of L3.6pl (L1empty, $L1_{KD\#1}$ and L1over) cells treated or not with 120 μM of Tranilast (TRL). *p < 0.05, **p < 0.005, ***p < 0.0005.

Supplementary Fig. 7:

a. Representative images of migration capacity (boyden chamber assay) for L3.6pl (L1empty, $L1_{KD\#1}$ and $L1^{over}$) cells treated or not with 25 nM of Batimastat (BB-94). **b.** Migratory potential of L3.6pl (L1empty,

$L1_{KD\#1}$ and L1over) cells treated or not with 25 nM of Batimastat (BB-94). **c.** Representative images of gelatin degradation for L3.6pl (L1empty, $L1_{KD\#1}$ and $L1^{over}$) cells treated or not with 120 µM of Tranilast (TRL). Nuclei were stained with Hoechst 33342 (blue) and green represents actin (Alexa Fluor™ 488 Phalloidin). **d.** Representative images of gelatin degradation for L3.6pl (L1empty, L1KD#1 and L1over) cells treated or not with 25 nM of Batimastat (BB-94). Nuclei were stained with Hoechst 33342 (blue), green represents actin (Alexa Fluor™488 Phalloidin) and red illustrates gelatin (Rodhamine). **e.** Invasive potential of L3.6pl (L1empty, L1KD#1 and L1over) cells treated or not with 25 nM of Batimastat (BB-94). **f.** Representative images of gelatin degradation for L3.6pl (L1empty, $L1_{KD\#1}$ and $L1^{over}$) cells treated or not with 25 nM of Batimastat (BB-94). Nuclei were stained with Hoechst 33342 (blue) and green represents actin (Alexa Fluor™ 488 Phalloidin). ***p < 0.0005.

Supplementary Fig. 8:

a. Boxplots showing the differential expression of *MMP2* in PDAC samples versus normal tissue (NP) in the TCGA dataset. **b.** Kaplan–Meier curves showing overall survival of PDAC patients, stratified according to the median value of *MMP2* expression. **c.** qPCR analysis for *MMP2* expression in #L3.6pl and #354 cells ($L1_{low}$ and $L1^{high}$). **d.** qPCR analysis for *MMP2* expression in #L3.6pl and #354 cells (L1empty, $L1_{KD\#1}$ and $L1^{over}$). **e.** Boxplots showing the differential expression of *MMP10* in PDAC samples versus normal tissue (NP) in the TCGA dataset. **f.** Kaplan–Meier curves showing overall survival of PDAC patients, stratified according to the median value of *MMP10* expression. **g.** qPCR analysis for *MMP10* expression in #L3.6pl cells ($L1_{low}$ and $L1^{high}$). **h.** qPCR analysis for *MMP10* expression in #L3.6pl and #354 cells (L1empty, $L1_{KD\#1}$ and $L1^{over}$). **i.** Representative H&E (upper) and Sirius Red (bottom) staining of pancreas from mice injected with L3.6pl cells, treated or not with TRL (50 mg/kg of mice). **j.** Representative histological sections of pancreas from mice injected with L3.6pl cells, treated or not with TRL (50 mg/kg of mice). Tumor sections were (immuno)stained for murine COL1A1. **k.** Representative histological sections of liver from mice injected with L3.6pl cells, treated or not with TRL (50 mg/kg of mice). Tumor sections were (immuno)stained for murine COL1A1. **l.** Representative histological sections of xenografts derived from #354 cells. Tumor sections were (immuno)stained for human murine COL1A1. *p < 0.05, **p < 0.005, ***p < 0.0005.

Supplementary Figure 1

a

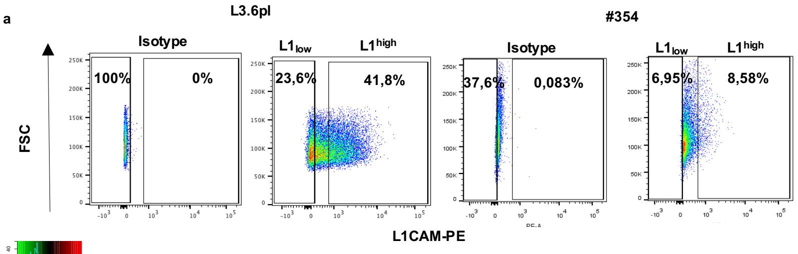

b

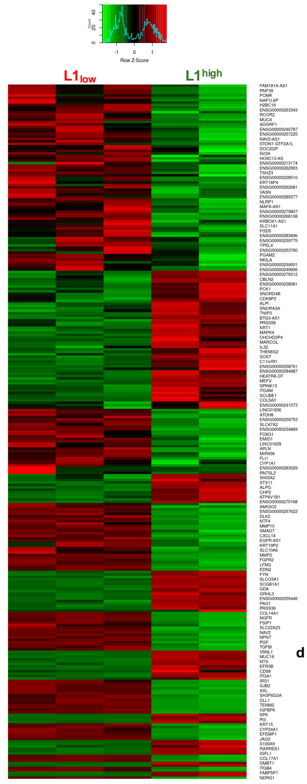

c

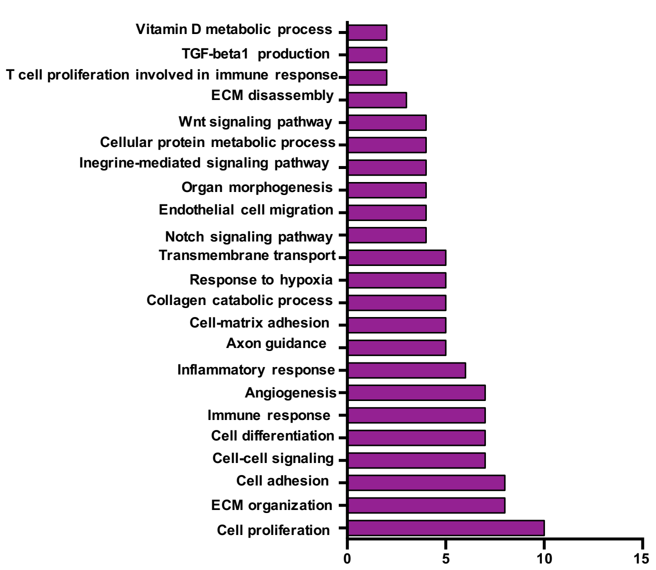

d

KEGG pathway	Genes	p-Value
Regulation of actin cytoskeleton	FGF18, ITGA1, ITGAM, PAK3, SCIN	9,7E-3
PPAR signaling pathway	FABP4, FABP5, PCK1	3,0E-2
Focal adhesion	FYN, COL5A3, ITGA1, PAK3	5,0E-2
Folate biosynthesis	ALP1, ALPP	5,65E-2
Axon guidance	FYN, L1CAM, PAK3	9,4E-2

e

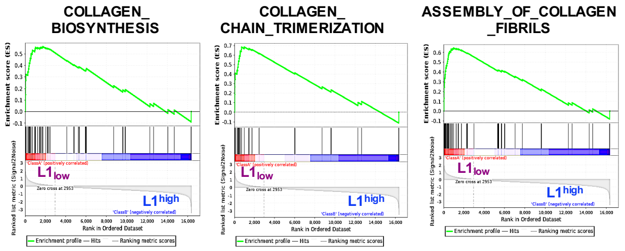

Supplementary Figure 2

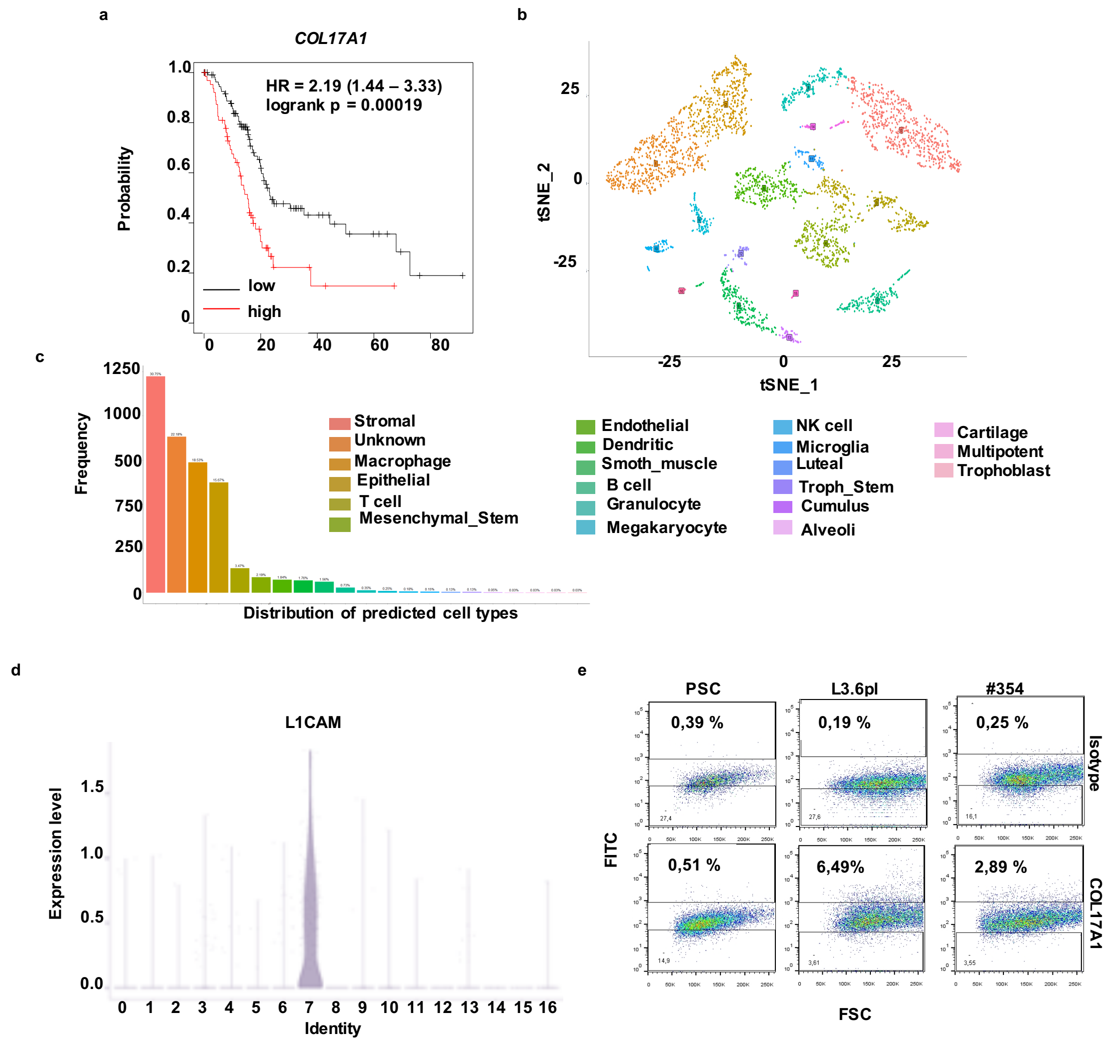

Supplementary Figure 3

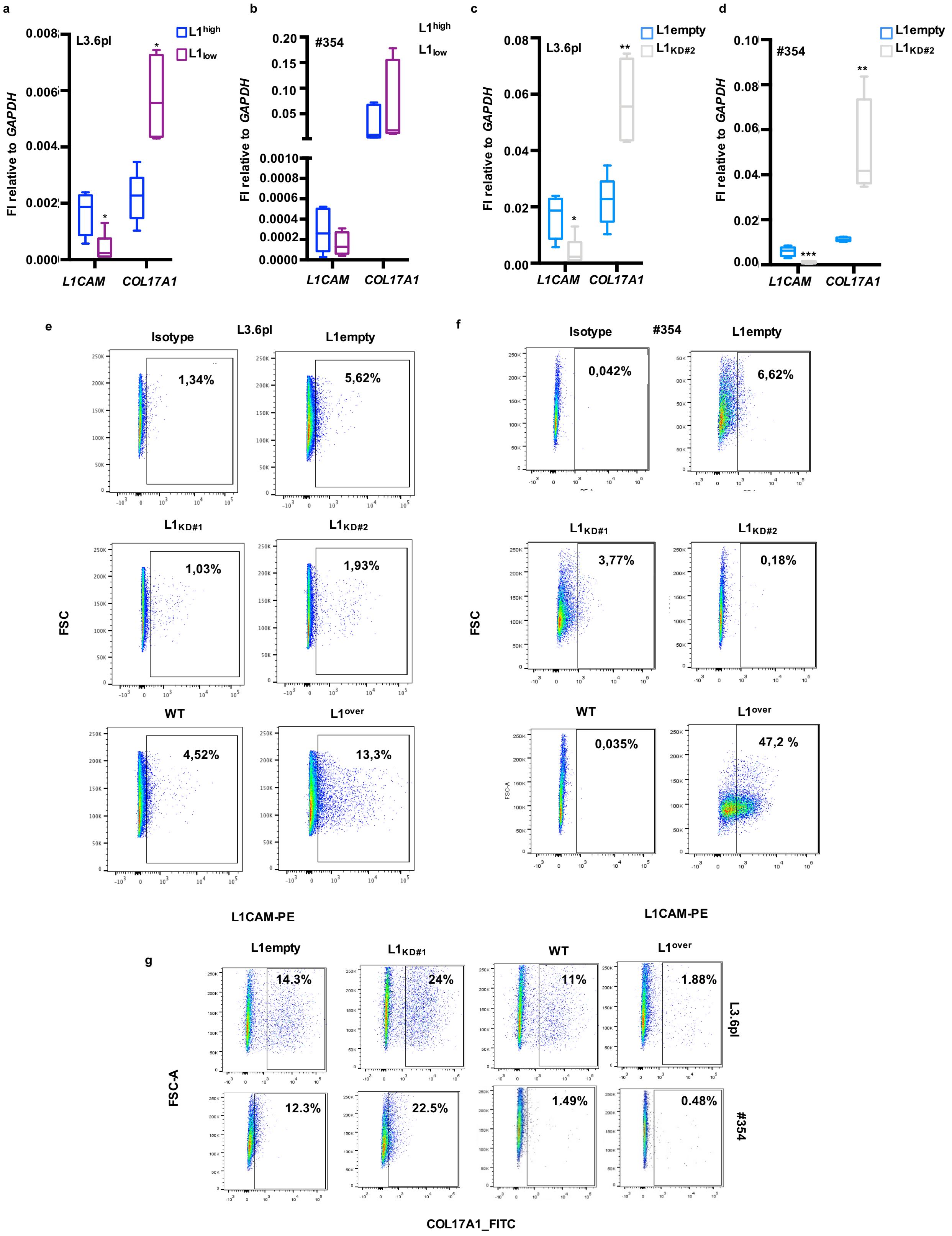

Supplementary Figure 4

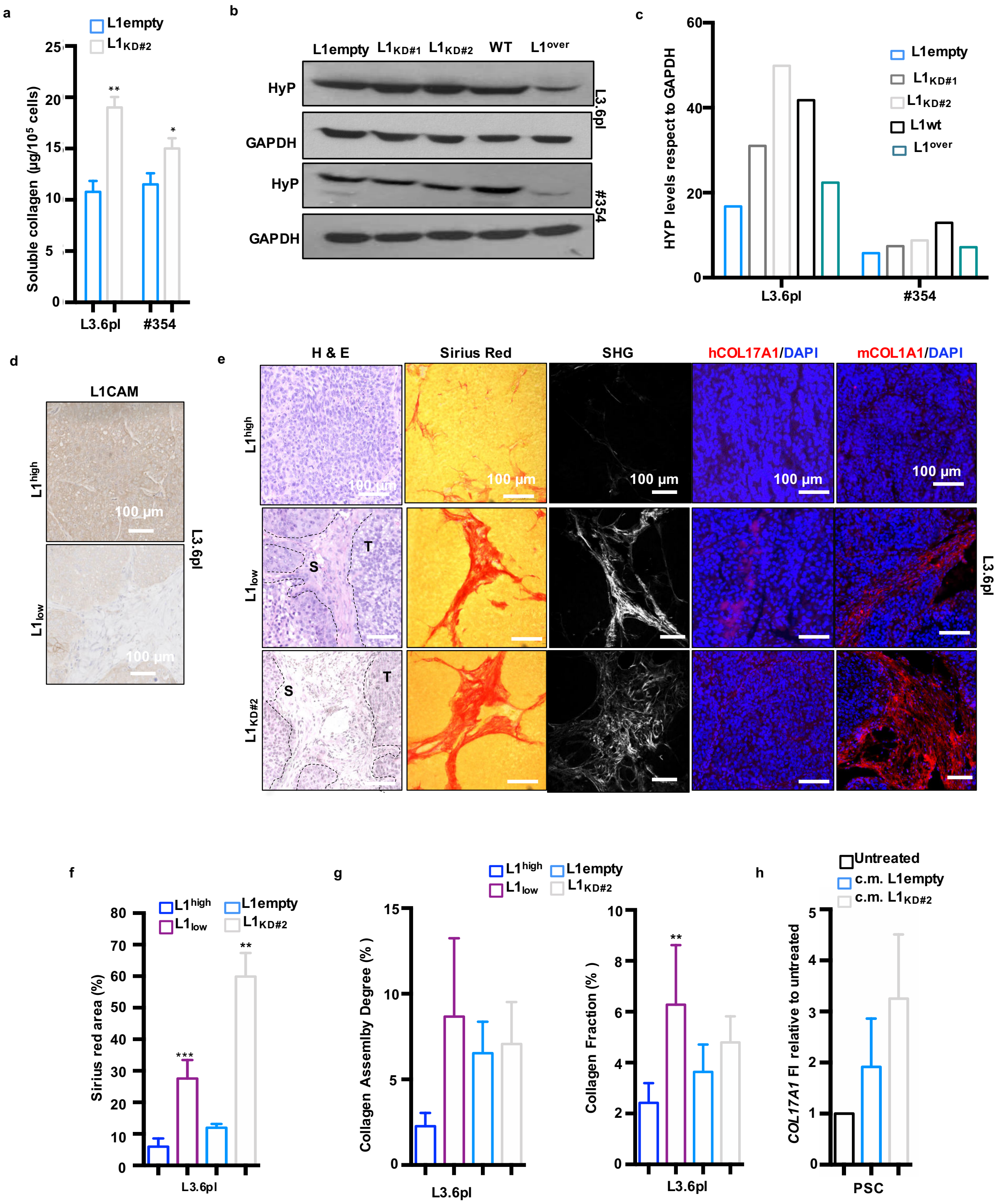

Supplementary Figure 5

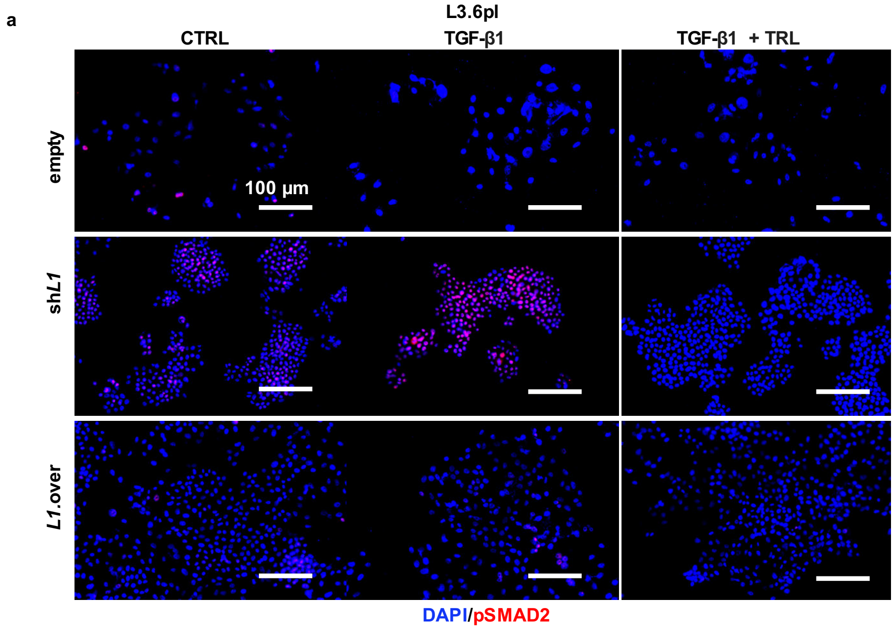

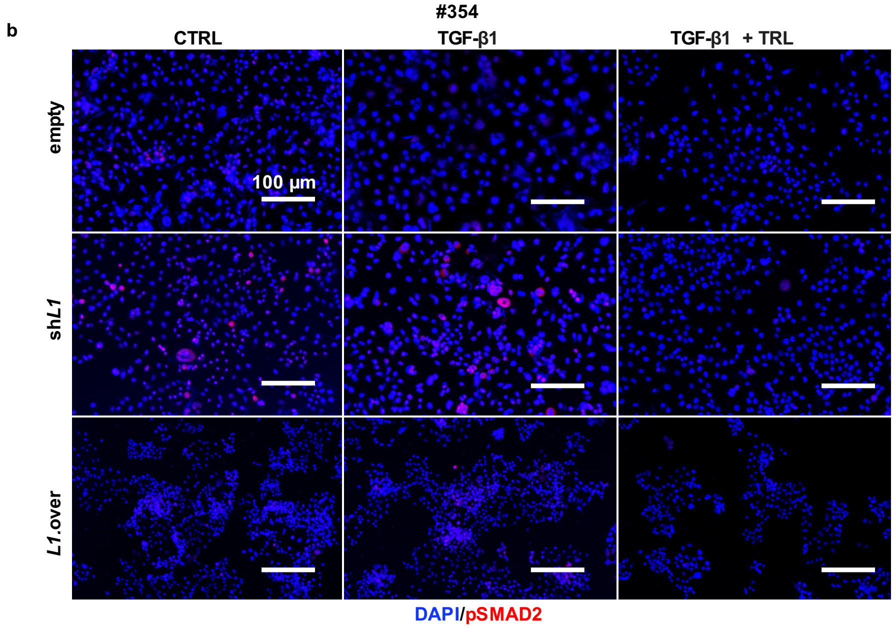

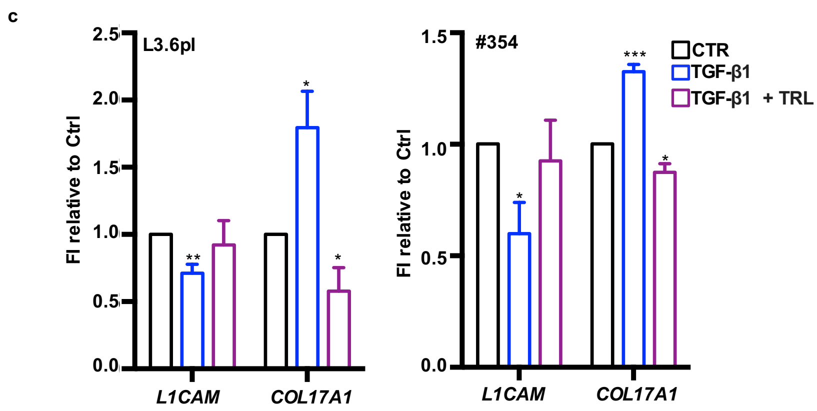

Supplementary Figure 6

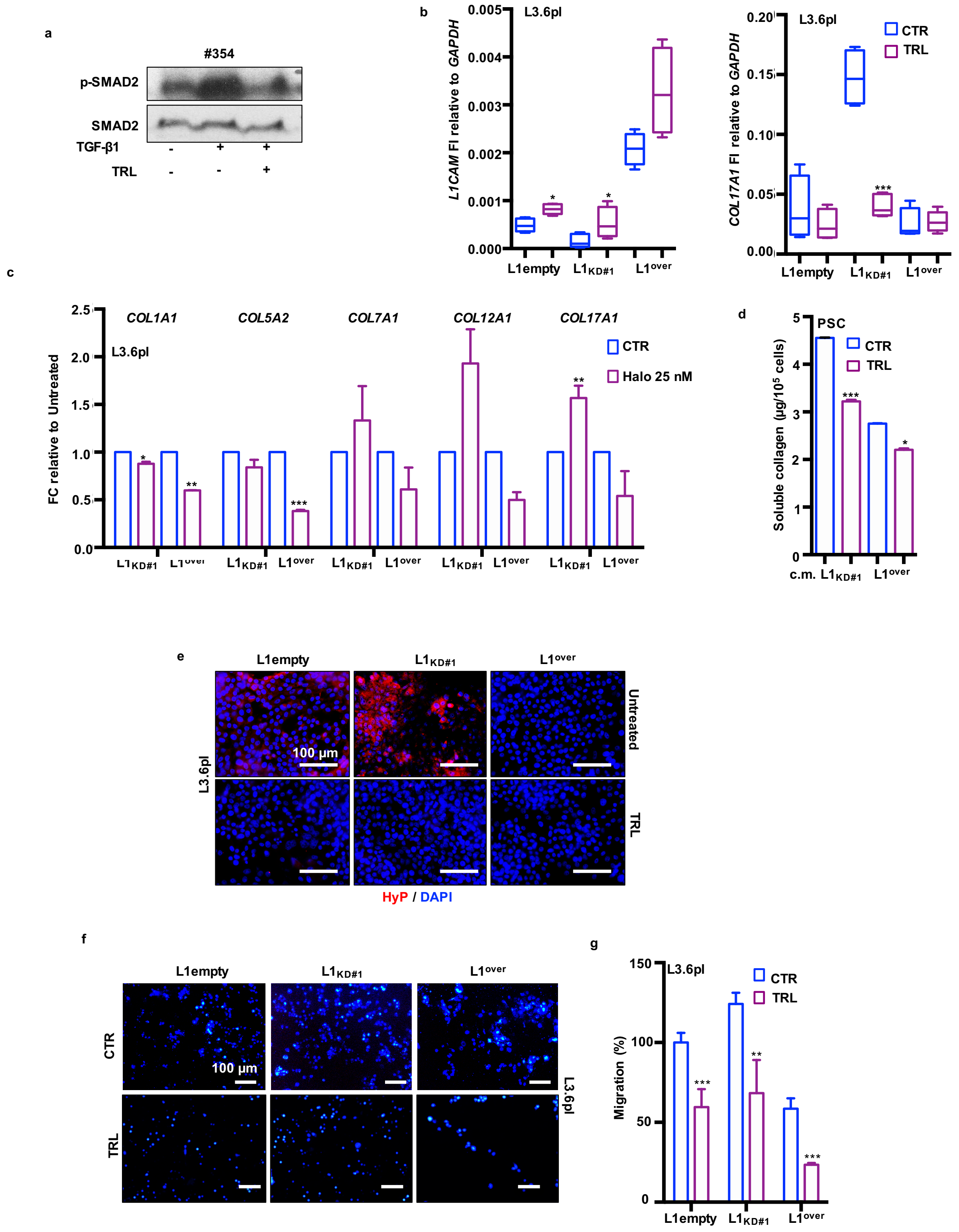

Supplementary Figure 7

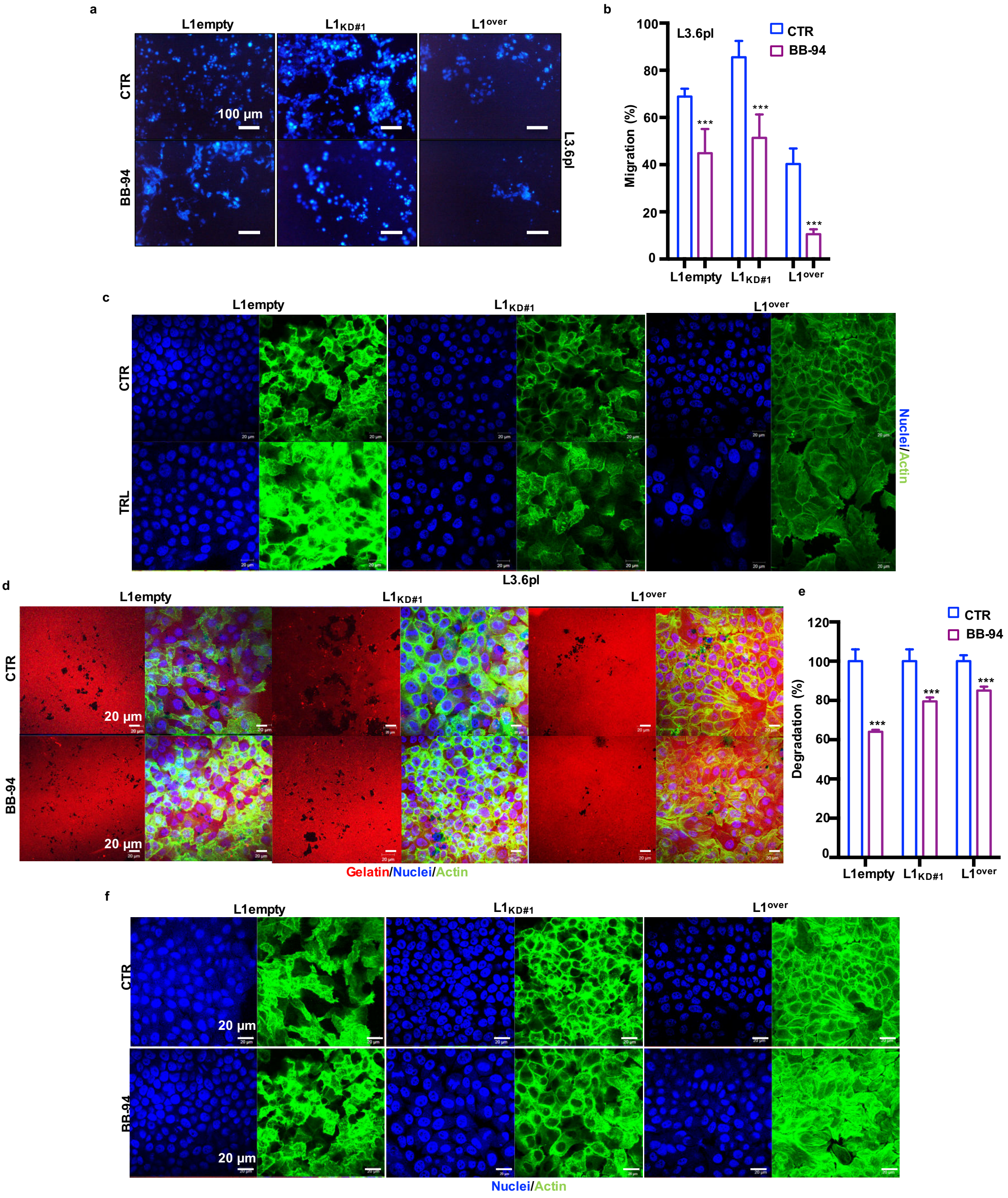

Supplementary Figure 8

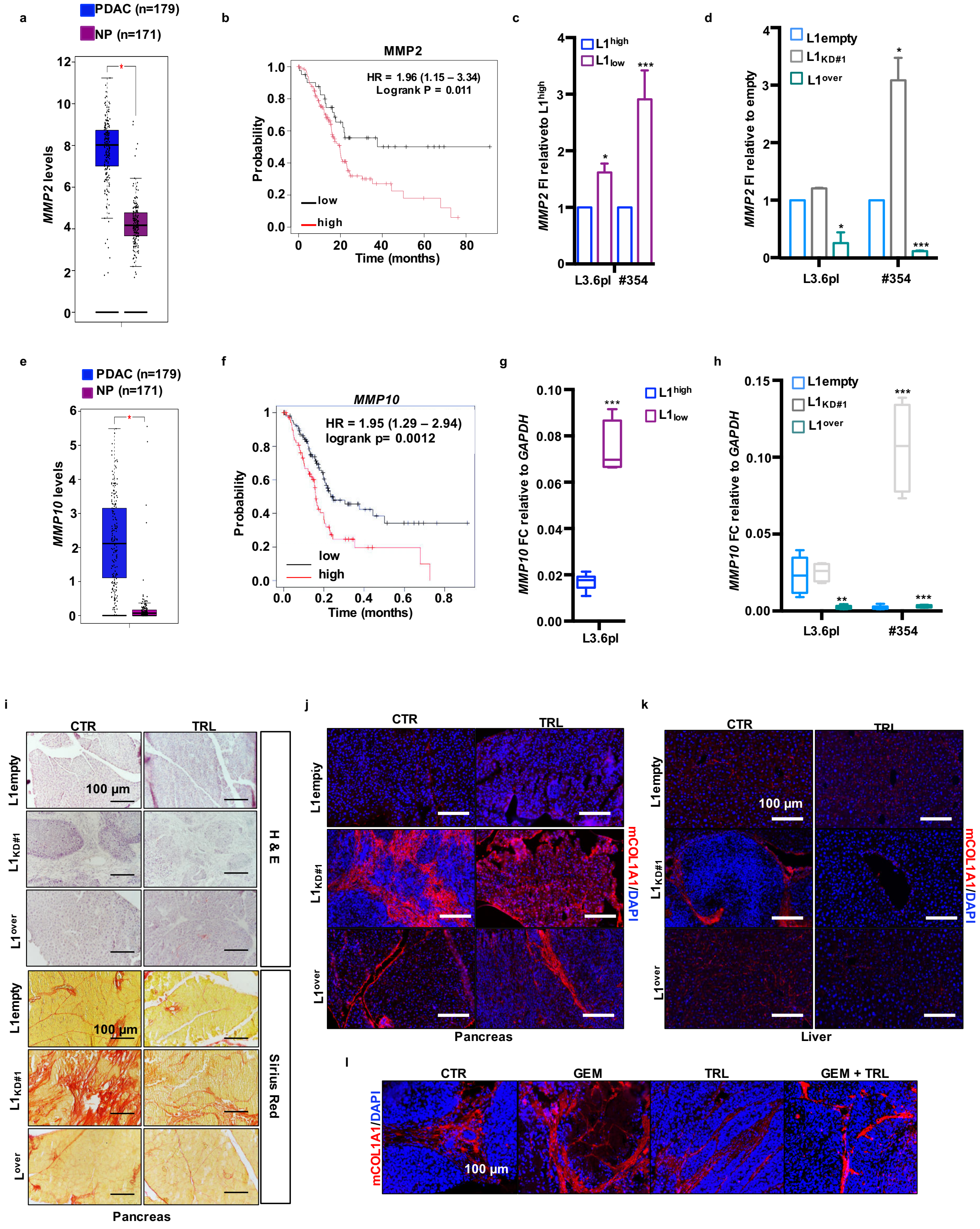

Supplement: Supplementary file 1 — Supplementary Data [file 41419_2025_7859_MOESM1_ESM.pdf]
