# Supplementary material for: TGF-β1-mediated downregulation of L1CAM in pancreatic ductal adenocarcinoma drives upregulation of collagen 17A1 and MMP2, facilitating tumor invasiveness and metastasis
Source: Cell Death Dis. 2025 Aug 6;16(1):592. doi: 10.1038/s41419-025-07859-8 (PMC12328658; doi:10.1038/s41419-025-07859-8)
Supplement: Supplementary file 2 — western blotting [file 41419_2025_7859_MOESM2_ESM.pdf]

#356.

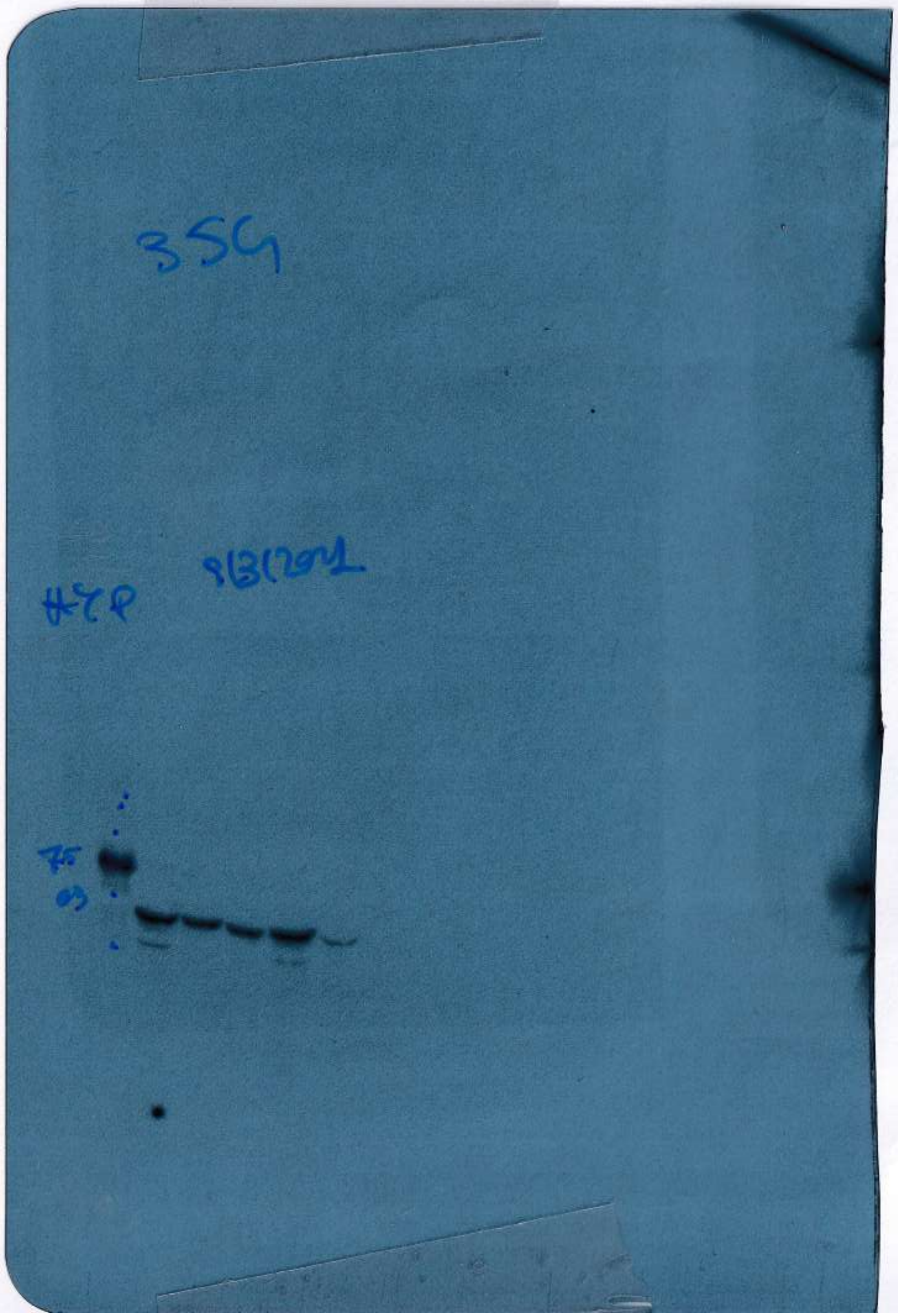

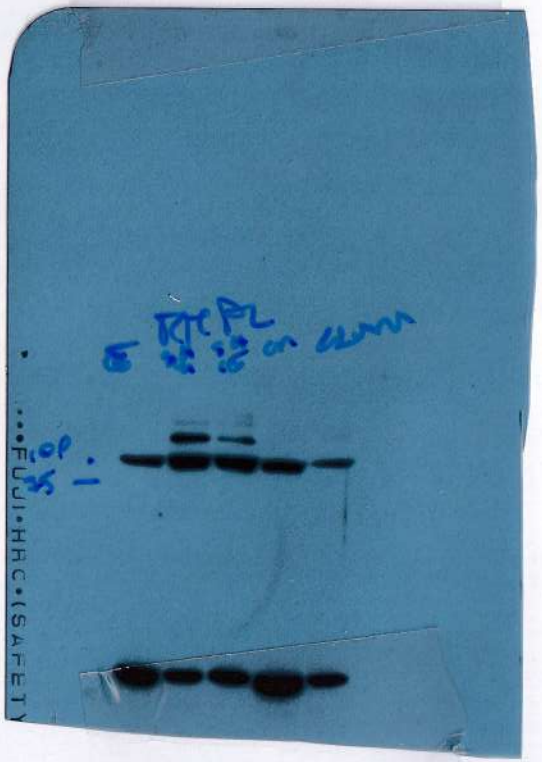

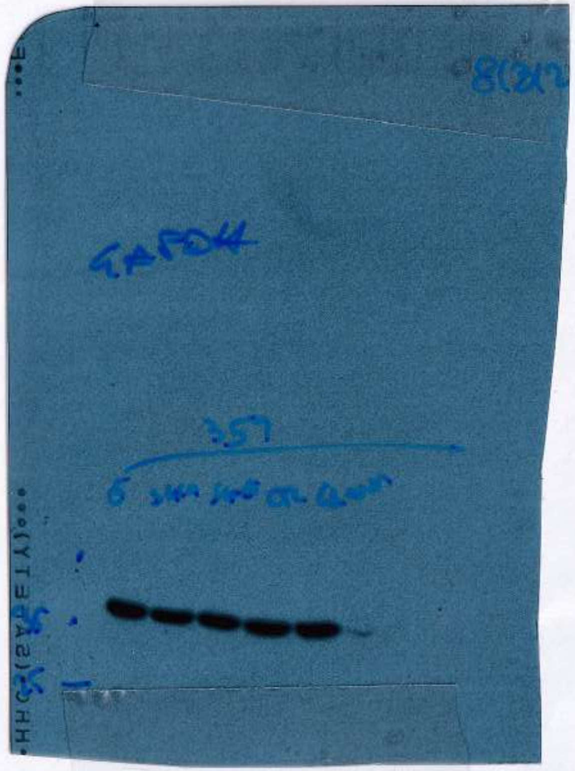

Revelage C3GAM: C36pl

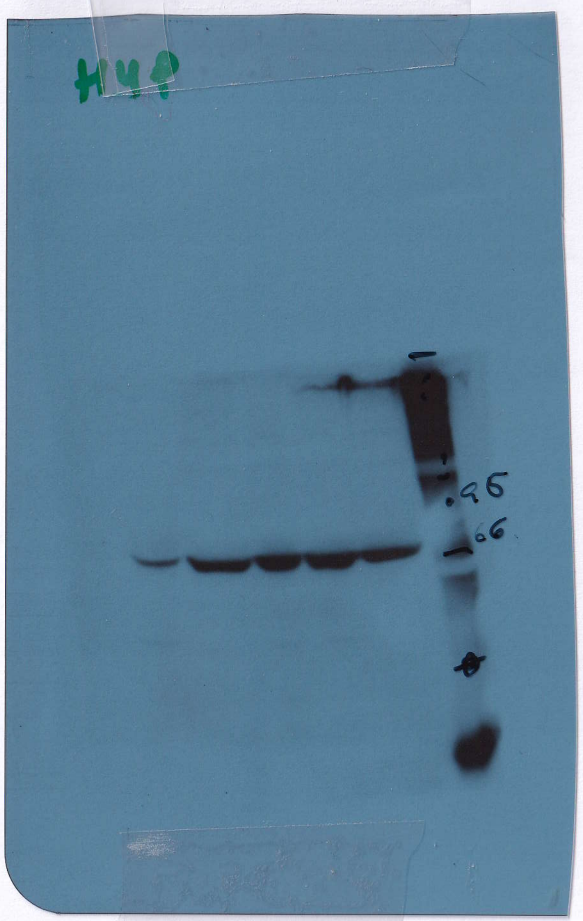

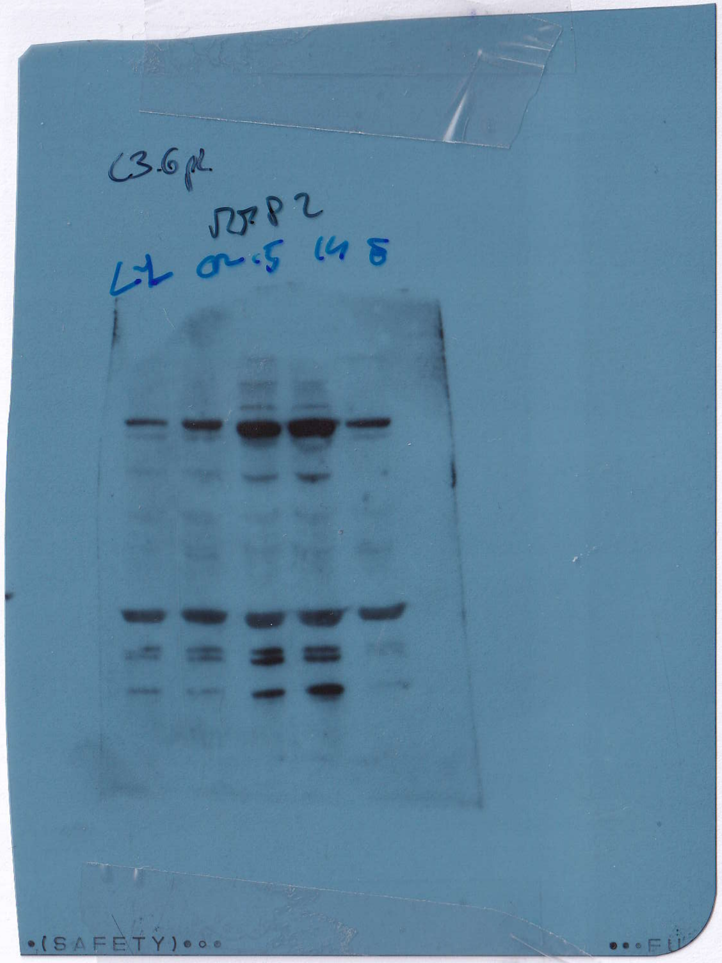

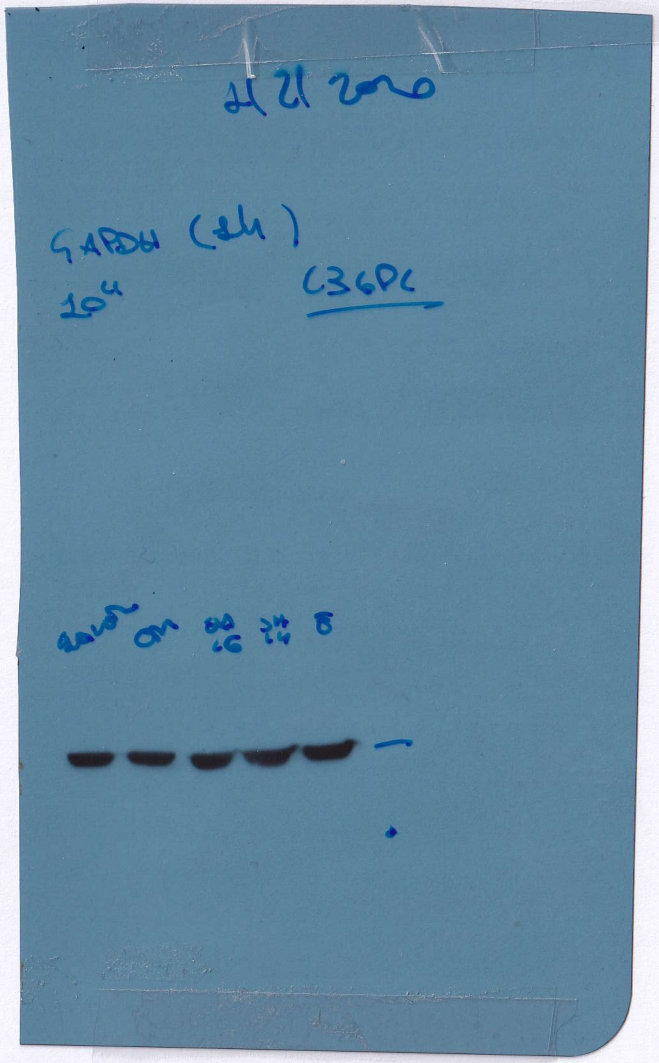

Revidaue [illegible]

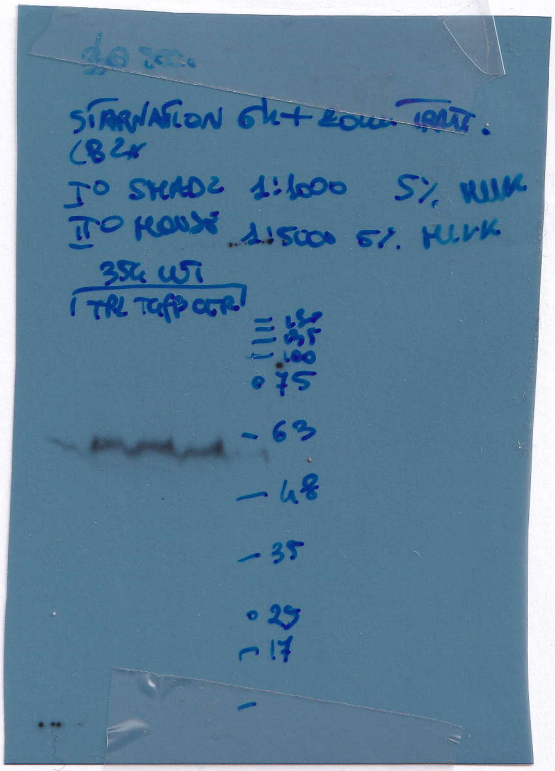
STARVATION 6h + [illegible]
CB2x
I° SMAD2 1:1000 5% MILK
II° MOUSE 1:5000 5% MILK
35u WT
TRL TGFβ CTR
75
63
48
35
25
17

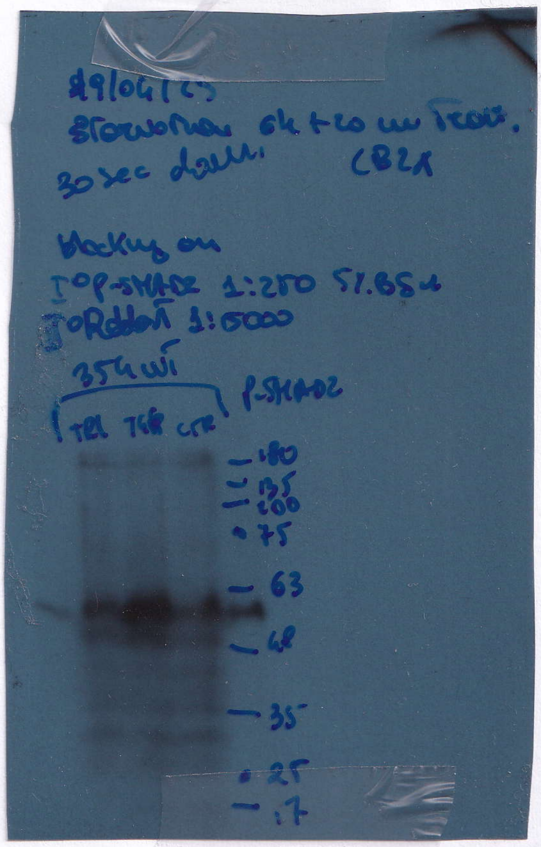
19/04/[illegible]
Starvation 6h + [illegible]
30 sec [illegible]
CB2X
blocking on
I° P-SMAD2 1:250 5% BSA
II° Rabbit 1:5000
35u WT
p-SMAD2
TRL TGFβ CTR
180
135
100
75
63
48
35
25
17
